# High-Resolution Regulatory Maps Connect Vascular Risk Variants to Disease-Related Pathways

**DOI:** 10.1161/CIRCGEN.118.002353

**Published:** 2019-02-20

**Authors:** Örjan Åkerborg, Rapolas Spalinskas, Sailendra Pradhananga, Anandashankar Anil, Pontus Höjer, Flore-Anne Poujade, Lasse Folkersen, Per Eriksson, Pelin Sahlén

**Affiliations:** 1 Science for Life Laboratory, Division of Gene Technology, School of Engineering Sciences in Chemistry, Biotechnology and Health, KTH Royal Institute of Technology, Solna, Sweden (Ö.Å., R.S., S.P., A.A., P.H., P.S.).; 2 Cardiovascular Medicine Unit, Department of Medicine, Center for Molecular Medicine, Karolinska Institutet, Stockholm, Sweden (F.-A.P., P.E.).; 3 Department of Bioinformatics, Technical University of Denmark, Copenhagen, Denmark (L.F.).

**Keywords:** coronary artery disease, gene, haplotype, inflammation, genomics

## Abstract

Genetic variant landscape of coronary artery disease is dominated by noncoding variants among which many occur within putative enhancers regulating the expression levels of relevant genes. It is crucial to assign the genetic variants to their correct genes both to gain insights into perturbed functions and better assess the risk of disease.

**Methods:**

In this study, we generated high-resolution genomic interaction maps (≈750 bases) in aortic endothelial, smooth muscle cells and THP-1 (human leukemia monocytic cell line) macrophages stimulated with lipopolysaccharide using Hi-C coupled with sequence capture targeting 25 429 features, including variants associated with coronary artery disease. We also sequenced their transcriptomes and mapped putative enhancers using chromatin immunoprecipitation with an antibody against H3K27Ac.

**Results:**

The regions interacting with promoters showed strong enrichment for enhancer elements and validated several previously known interactions and enhancers. We detected interactions for 727 risk variants obtained by genome-wide association studies and identified novel, as well as established genes and functions associated with cardiovascular diseases. We were able to assign potential target genes for additional 398 genome-wide association studies variants using haplotype information, thereby identifying additional relevant genes and functions. Importantly, we discovered that a subset of risk variants interact with multiple promoters and their expression levels were strongly correlated.

**Conclusions:**

In summary, we present a catalog of candidate genes regulated by coronary artery disease–related variants and think that it will be an invaluable resource to further the investigation of cardiovascular pathologies and disease.

## Abstract 2

Supplemental Digital Content is available in the text.

Coronary artery disease (CAD) labels medical problems of the circulatory system (heart, blood vessels, and arteries) often because of build-up of fatty cell debris (plaques) deposited inside the blood vessels. It is the leading cause of disability and death globally.^1^ Atherosclerosis, the main underlying mechanism leading to the acute events of CAD, is characterized by a lipid driven chronic inflammation of the arterial intima, a process that includes all major cells in the vascular wall, that is, endothelial, smooth muscle, and inflammatory cells. The acute complication of atherosclerosis, such as myocardial infarction and stroke is because of rupture of the fibrous cap with subsequent thrombus formation that totally or partially occludes the vessel and thereby stops the nutrient-rich blood flow. Traditional risk assessment methods based on age, sex, smoking, diabetes mellitus, hypertension, and dyslipidemia tracks the disease incidence well but underestimates its occurrence because almost half the population classified as low or intermediate risk end up developing cardiovascular disease^2–4^ as these methods fail to inform on the underlying pathological processes that may have been going on for years.^5^ In addition, ethnic differences in cholesterol and blood lipid levels complicate the assessment of the individual risk of disease.^6^ Heritability for CAD is estimated between 40% and 60% based on twin and family studies; therefore, genetic risk contributors at play can be utilized in its early diagnosis and treatment.^7^

Genome-wide association studies (GWAS) have emerged as an important tool in the search for disease-causing genomic variants.^8^ CAD-specific and other atherosclerosis-related indications have been addressed by large GWAS meta-analyses enabled by consortia, such as the CARDIoGRAMplusC4D (Coronary Artery Disease Genome wide Replication and Meta-analysis [CARDIoGRAM] plus The Coronary Artery Disease [C4D] Genetics)^9^ and the MEGASTROKE (International Stroke Genetics Consortium) consortium.^10^ At its current state, just over 300 independent variants explain 21% of CAD heritability.^11^ According to GWAS, a locus on chromosome 9p21 has the strongest association signal.^12,13^ Although it is established that the risk allele is associated with formation and progression of plaques but not with their rupture,^14,15^ the mechanistic understanding of the conferred risk by these loci remains elusive.^16–18^ Pathways such as cholesterol and triglyceride metabolism, blood pressure, inflammation, vascular proliferation and remodeling, nitric oxide signaling, vascular tone, extracellular matrix integrity, and axon guidance and signaling are also enriched for target genes of GWAS variants.^17–20^

GWAS studies do not in themselves provide functional insight for the large subset of hits that are noncoding^21,22^: only one-third of the time a variant affects the expression level of its nearest gene, highlighting the limitations of the nearest gene assignment approach.^23,24^ The target gene mappings can be refined using various layers of genome annotation information as well as gene expression profiles. To alleviate the problem of complex linkage structures between variants, vast amounts of public datasets of epigenetic marks and transcription factor binding profiles used to help prioritize the causal/functional variant.^25–27^ Expression quantitative trait loci analyses based on gene expression and genotype datasets are also used to locate potentially functional variants that are in linkage disequilibrium (LD) with top association variants.^28–30^

Pathway or gene set–based approaches using canonical pathways and gene ontology (GO) terms goes beyond single variant-based analyses and investigate the combined effect of multiple disease/trait variants on biological functions in terms of the perturbations on pathways or cellular processes.^31–35^ Such pathway-based analyses revealed the functional GWAS variants in cases, such as Crohn disease,^36^ multiple sclerosis,^37^ schizophrenia,^38^ and breast cancer.^39^ Functional gene sets built using coexpression, and protein-protein interaction datasets are also used successfully to interpret the GWAS variants.^40–42^

Many promoters require regulatory elements called enhancers to drive and regulate gene expression. Enhancers can be located at long distances from their cognate promoters and brought into contact via chromatin looping. Many enhancers carry tissue-specific epigenetic marks such as H3K4me1 or H3K27Ac, facilitating their discovery, however, not providing information on gene(s) they act on. Studies of the chromatin interaction landscape were revolutionized by the invention of chromosome conformation capture coupled with next-generation sequencing (Hi-C) enabling the study of genome structure and folding. Combining Hi-C with sequence capture (HiCap), the improvement in resolution required to study individual promoter-enhancer interactions can be obtained.^43^ A recent study also used high-resolution chromatin conformation capture to obtain promoter-anchored regulatory landscape of induced pluripotent stem cells and induced pluripotent stem cell–derived cardiomyocytes, providing a valuable resource for the cardiovascular biology.^44^

In this study, we used HiCap on 3 cell types relevant for vascular diseases, particularly atherosclerosis and aortic diseases, to discover novel biological processes and pathways related to onset and pathology of the disease. We utilized chromatin contacts of promoters to GWAS variants or those that are in LD to assign potential target genes.

## Methods

The authors declare that all supporting process data are available within its Data Supplement. The raw data files (fastq and bam files) that support the findings of this study are available from the corresponding author on reasonable request. This study does not involve animal studies. The study was approved by the Human Research Ethics Committee at Karolinska Institutet (application number 2006/784-31/1 and 2012/1633-31/4), Stockholm, Sweden; written informed consent was obtained from all the patients according to the Declaration of Helsinki, and methods were performed in accordance with relevant guidelines. Please see Data Supplement for detailed method descriptions.

## Results

**Figure 1.**
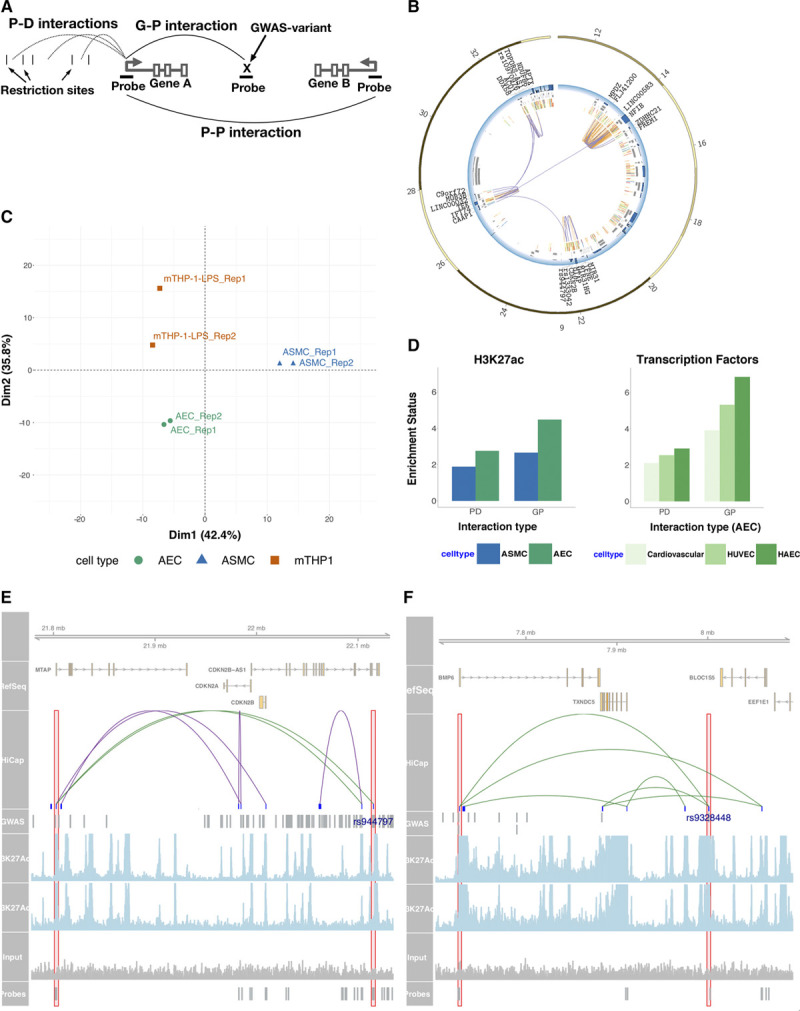
**Promoter interacting regions were enriched for regulatory elements. A**, Three types of Hi-C with sequence capture-established interactions; promotor (P)-distal (D), P-P and genome-wide association studies (GWAS)-P (G-P), respectively; for the Gene A promoter. In P-D is analyzed probed promoters’ interaction with D element (DEs); the latter separated by restriction sites. In P-P and G-P both ends of the interaction are probed. **B**, The largest connected subgraph in chr9 in aortic endothelial cell (AEC). There are 22 promoters, 3 GWAS SNPs and 271 DE (only those overlapping with H3K27ac marks are included). The blue track represents gene expression levels, gray boxes represent transcripts, and innermost layers represent H3K27Ac marks (2 replicates). Purple arcs represent G-P or P-P, and orange arcs represent P-D interactions respectively. **C**, Principal component analysis of P-D interaction of genes not expressed in none of the cell types **D**, Overlap enrichment relative to a segment-length and distance-from-P controlled random set. The AEC P-D and G-P datasets were overlapped with general cardiovascular, HUVEC, and HAEC transcription factor marker data from ChipAtlas. **E**, Three hundred and fifty-seven kilobase region containing MTAP-rs944797 interaction and (**F**) 393 kb region containing BMP6-rs9328448 interaction visualized using Gviz package.^46^ Overlap with H3K27Ac marks are shown in the lower panes as well as the signal from the input chromatin. P-D (including P-G) and P-P interactions are colored as green and purple respectively. ASMC indicates aortic smooth muscle cells; BMP6, Bone Morphogenetic Protein 6; HAEC, human aortic endothelial cell line; HUVEC, Human umbilical vein endothelial cells; LPS, lipopolysaccharides; MTAP, Methylthioadenosine Phosphorylase; mTHP, macrophage–THP-1; and SNP, single nucleotide polymorphism.

Using high-resolution chromatin interactions, we mapped genomic interaction of promoters and variants associated with traits and conditions related to cardiovascular diseases in particular coronary artery and aortic diseases. Three cell types for the investigation are human aortic endothelial cells (AEC), human aortic smooth muscle cells (ASMC) and macrophage–THP-1 cells (mTHP-1–lipopolysaccharide) stimulated with lipopolysaccharide for 2 hours. For AEC, we obtained 2 technical replicates from 2 individuals; the technical replicates were pooled, and the 2 individuals were held separate and constituted biological replicates. For ASMC and mTHP-1–lipopolysaccharide cells, 2 technical replicates were obtained. We used HiCap using a probe set targeting 21 479 promoters and 3950 variants (Table I in the Data Supplement). There were 199, 300, and 289 million read pairs uniquely mapped to probes in AEC, ASMC, and mTHP-1–lipopolysaccharide experiments, respectively (Table II in the Data Supplement). We made a distinction between interactions between promoters (promoter-promoter or Prom-Prom) and those between promoters and elsewhere in the genome (promoter-distal or Prom-Dist). For the sake of clarity, we also defined interactions between disease/trait associated variants and promoters (GWAS-Promoter or GWAS-Prom; Figure 1A).

We called interactions using HiCapTools requiring each interaction present in both replicates, and *P* value cutoffs deployed yielded interaction sets of sizes 69,753 (AEC Prom-Dist), 38 759 (ASMC Prom-Dist), 19 920 (mTHP-1–lipopolysaccharide Prom-Distal), and 5671 (AEC Prom-Prom) and 4293 (ASMC Prom-Prom), and 1698 (mTHP-1–lipopolysaccharide Prom-Prom), respectively (Table IIIA, IIIB, and IIIC and Methods in the Data Supplement).^45^ Importantly, we were able to detect many long-range (>500 kb) interactions across the 3 cell types (Figure I in the Data Supplement). Equally important, the distal elements (DEs), as well as the interacting promoters were short; average length being 749 and 776 bases, respectively. In total, the interaction datasets covered around 3.4% of the genome. Most promoters (65%) were found to interact with <5 distal regions, whereas the interactome of extreme hub-promoters contain several hundred DEs (Figure IB through ID in the Data Supplement). We identified several interconnected units of promoters and enhancers; Figure 1B displays the largest connected subsection of chromosome 9 (ie, giant component). Interestingly, 2 cardiovascular disease (CVD) associated GWAS single nucleotide polymorphisms (SNPs) in chr9p21 region (rs1333042 and rs944797) were part of this network.

We profiled expression levels of genes in AEC, ASMC, and mTHP-1–lipopolysaccharide cell lines using RNA sequencing (Table IV in the Data Supplement). Utilizing principal component analysis, we show that the promoter-interaction profiles of individual cell types are specific and can separate individual cell types independent of gene expression information (Figure 1C, Figure III in the Data Supplement).

### Promoter-Interacting Distal Elements Were Enriched for Functional Elements

Promoter-interacting DEs were previously shown to be highly enriched for enhancer marking features. To confirm that is also the case for this study, we overlapped DEs with H3K27ac enriched regions obtained through chromatin immunoprecipitation-seq in the same cells, as well as relevant DNaseI and transcription factor binding datasets from the ChipAtlas (list of public datasets can be found in Table V in the Data Supplement and www.chip-atlas.org). Our interactor sets were indeed enriched relative to size-controlled and genomic context–controlled random sets, and the enrichment was stronger for the better matching cell types (Figure 1D, Figure IVA through IVD and Feature Enrichment Analysis in the Data Supplement). Interestingly, enrichment levels for the GWAS-Prom set were much higher in AECs and ASMCs (Figure 1D, Figure IVA through IVD in the Data Supplement). Furthermore, promoters interacting with DEs carrying H3K27ac marks were expressed at higher levels as expected (Figure IVE in the Data Supplement).

Figure 1E and 1F show 2 examples of promoter interactions (MTAP-rs944797 and BMP6-rs9328448) where the interactor overlaps with both CVD GWAS variants and cell-specific H3K27Ac enhancer marks.

### Promoter Interacting GWAS Variants Were Often Contained Within Regulatory Elements

**Figure 2.**
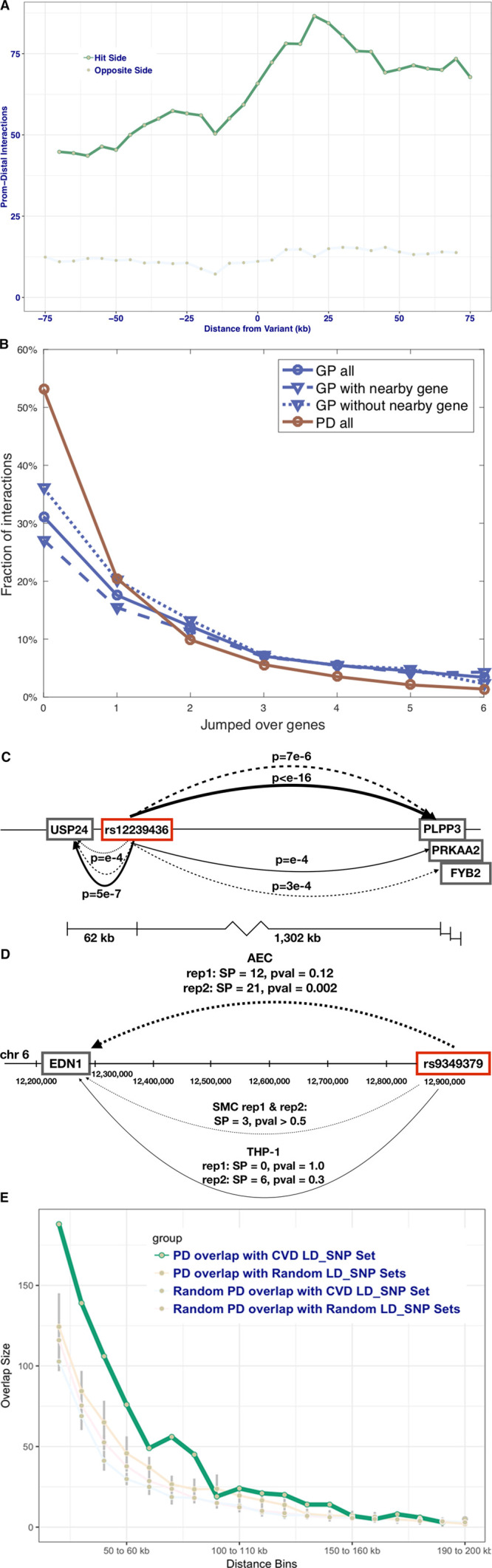
**HiCap can inform on regulatory potential of variants in LD with risk variants. A**, The aortic endothelial cell (AEC) Prom-Dist (PD) set was searched for interactions between the promoter and distal elements close to a variant interacting with the same promoter (green curve). The comparison was made relative to a site at the same distance from the promoter it but located at the other side of it (light blue curve). The latter is nearly a horizontal line as expected, whereas the blue curve strongly deviates from that at distances not too far from the variant site. The bin size used to count interactions is 5 kb. **B**, Variants in the AEC, aortic smooth muscle cell (ASMC) and macrophage–THP-1 (mTHP-1)-lipopolysaccharide (LPS) merged genome-wide association studies (GWAS)-Prom (GP) set and their interaction preferences with genes at distance 0 (no gene-jumping), 1 (nearest gene is jumped over), etc. Distal regions in the corresponding PD set are shown for reference. The GP interaction set is further split in equal-sized halves depending on the variants’ distance to its nearest gene (GP without and with a nearby gene, respectively). **C**, The coronary artery disease (CAD)–related variant rs12239436 (red box) interacts with 62 kb distant gene *USP24* (gray box) in AEC (dotted line), ASMC (dashed,) and mTHP-1–LPS (solid). Strengths of interactions are represented with the *P* value recorded and indicated by arrow thickness. Our result set further include strong interactions with the 1.3 Mb distant previously CAD associated gene *PLPP3* (also known as *PPAP2B*). Less significant interactions with nearby *FYB2* (ASMC) and *PRKAA2* (mTHP-1–LPS) are potentially bystanders. **D**, Earlier reported interaction between variant rs934937 and the *EDN1* gene is, to a varying degree, present in both AEC patients. Not so in less relevant tissues ASMC and mTHP-1–LPS. **E**, Comparison of overlaps between PD dataset (AEC) vs 100 matched random datasets and CVD_GWAS and matched SNP datasets (see methods) shows that there is an enrichment for variants in linkage disequilibrium (LD) with CVD_GWAS found in PD dataset when the genomic distance between SNP and its LD proxy is ≤80 kb. No such enrichment was seen for random PD datasets vs real or random SNP sets. chr indicates chromosome; CVD, cardiovascular disease; SMC, smooth muscle cells; SNP, single nucleotide polymorphism; and SP, supporting pair.

We next turned our attention to variants associated with vascular disease phenotypes according to GWA studies and asked if any variants or in LD with those are contained within DEs found in this study. First, we took all single nucleotide variants associated with cardiovascular disease resulting in 3814 SNPs (minimum *P* value for association is 10^-6^), which we called CVD_GWAS (cardiovascular disease GWAS; Table VIA in the Data Supplement). As previously shown, a substantial portion of GWAS variants are themselves located within potential regulatory regions. We, therefore, targeted a subset (723, 19%) of these variants using probes to increase the probability of obtaining a signal without the need for deep sequencing (Table IVB in the Data Supplement). Of those 723 targeted GWAS variants, 295 (41%) interacted with at least 1 promoter in at least 1 cell type, constituting the GWAS-Prom dataset. We assigned 423 target genes to 295 variants (54% interacting with only one promoter). To rule out the possibility that GWAS-Prom hits occur merely a result of the probing of variants, we investigated the corresponding Prom-Dist set, that is, we studied the same promoters and their interactions with nonprobed distal regions. There should be vastly more Prom-Dist hits close to the variant site, than to a site on the same distance from the promoter but at the other side of it (hence keeping the distance from the probed feature the same). This is indeed the case as shown in Figure 2A (Interaction Density Comparison of Promoter-GWAS Hits in the Data Supplement).

A large fraction of GWAS-Prom interactions spanned distances above 500 kb (Figure I in the Data Supplement). Consequently, many of the GWAS variants (68%) are found to interact with nonclosest genes thus jumped over by the interaction loop formed (Figure 2B). However, only 43% of the Prom-Dist interactions involved nonclosest gene.

Whole groups of genes frequently interact with the same GWAS variant. Promoters of genes *USP24 (Ubiquitin Specific Peptidase 24), PLPP3 (Phospholipid Phosphatase 3), PRKAA2*, and *FYB2* (FYN Binding Protein 2) thus share a putative enhancer containing variant rs12239436. The rs12239436-*PLPP3* interaction is particularly interesting because of its large 1.3 Mb distance, as well as the fact that *PLPP3* was already identified as a CAD disease risk gene (Figure 2C).

The GWAS variant rs9349379, associated with 5 vascular diseases, was recently shown to regulate expression of the endothelin-1 gene. We see this interaction in 1 of the 2 AEC investigated individuals. Interestingly, the individual with the stronger interaction is heterozygous for rs9349379 (A/G), whereas the other individual is homozygous reference (A/A). In ASMC, where there is no interaction, rs9349379 variant is homozygous alternative (G/G; Figure 2D).

### Discovery of Target Genes of GWAS Variants Using Shared Haplotype and Interaction Information

To detect further interactions of CVD_GWAS variants with promoters, we looked at the fraction of DEs containing such variants. Of the 3814 associated variants in the CVD_GWAS, there were 216 (5.7%) variants within unique DEs. One complication of GWA studies is that the association signal many GWAS variants possess is because of their sharing of haplotype with the functional variants. If the functional variant can indeed modulate a distal promoter via looping, it should also be possible to locate it in our DE datasets. We, therefore, looked at the fraction of DEs that contain variants that are in LD with those in CVD_GWAS. Because of the sheer number of SNPs in LD, we devised a double randomization scheme to assign statistical significance to the observed overlap between LD SNPs and DE datasets using both size- and context-matched random interaction datasets and random SNP datasets matched with respect to allele frequencies and surrounding LD structure of the real set (Double Randomisation for LD SNP Overlap in the Data Supplement). We obtained LD and allele frequencies from 1000 Genomes (Phase 3 v5) using European population and set LD threshold of 0.8. Figure 2E shows that the DE dataset is enriched for SNPs in LD with CVD_GWAS that are within 80, 20, and 30 kb in AEC, ASMC, and mTHP-1–lipopolysaccharide cells, respectively, beyond which no enrichment can be seen (Figure VA and VB in the Data Supplement). There were 559 SNPs in LD with variants in CVD_GWAS located within DE fragments (Prom-Dist_LD). DEs carrying either the GWAS variant themselves or those in LD showed higher enrichments for open chromatin, transcription factor binding sites and enhancer marks compared with the entire DE set, supporting their potential for expression modulation (Figure VI in the Data Supplement).

**Figure 3.**
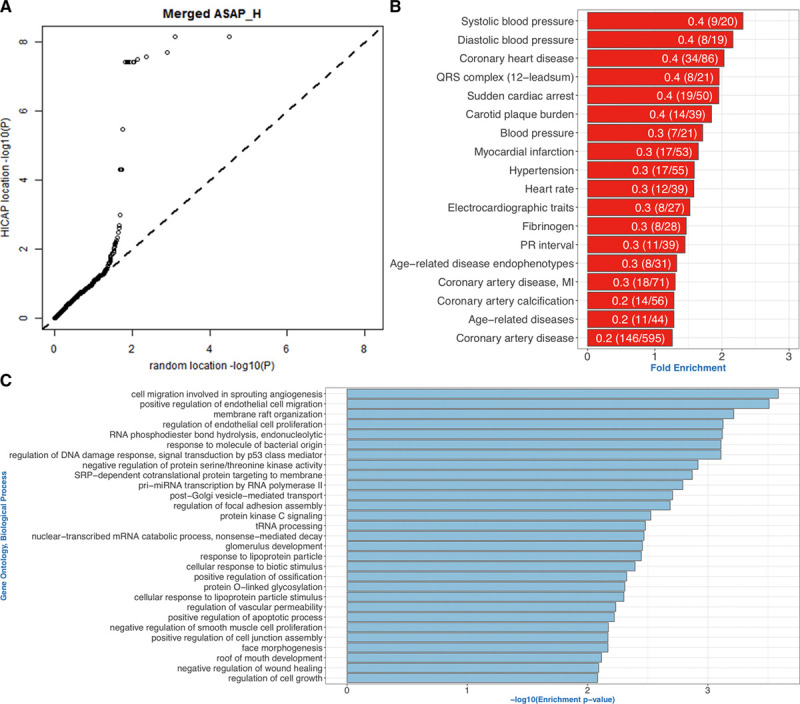
**Assigned target genes of CVD variants were enriched for pathways relevant for vascular pathologies. A**, Expression quantitative trait loci contained in the merged CVD genome-wide association studies (GWAS)-Promotor (prom), Prom- distal (Dist) and Prom-Dist_ linkage disequilibrium (LD) datasets plotted vs a size and distance-corrected random set. Deviation from diagonal is present among approximately 30% of the data. **B**, GWAS traits that are overrepresented in the interaction datasets. Only traits containing at least 14 variants were taken forward. Fold enrichment is calculated by dividing the actual number of trait variants in the interaction dataset to that of expected (fraction of trait variants in the full trait set). The bar labels denote the fraction of variants found in the interaction datasets. **C**, Gene ontology (GO) term enrichment analysis of genes interacting with variants or those in LD with CVD_GWAS set using TopGO package. GO terms enriched using only nearest genes to the variants are not reported. Terms with >5 genes and enrichment score >0.05 were not included. ASAP_H indicates The Advanced Study of Aortic Pathology, heart tissue; HiCap, Hi-C with sequence capture; MI, myocardial infarction; PR interval, the period, measured in milliseconds, that extends from the beginning of the P wave (the onset of atrial depolarization) until the beginning of the QRS complex; and Pri-miRNA, primary transcript of micro RNA.

The expression quantitative trait loci technique was deployed to examine the expression modulation capacity of variants contained in DEs. The GWAS-Prom set was extended with Prom-Dist hits and likewise selected hits in LD with those (Prom-Dist_LD). The comparison was performed relative to the aforementioned size- and distance-controlled random set and yielded the Q-Q plot presented in Figure 3B (merged set) and Figure VIIA through VIIC in the Data Supplement (GWAS-Prom, Prom-Dist, and Prom-Dist_LD separate). The deviation from the diagonal is striking and concerns not only extreme cases but large fractions of the entire sets.

Only 30% of the DEs containing these variants interacted with the closest gene and the average interaction distance is 301 kb (Table VI in the Data Supplement). In terms of trait categorization, variants for 167 of the 247 traits (0.67) related to CVD in EBI GWAS catalog was found, Figure 3C lists overrepresented traits in our dataset. We also compared enrichment of promoter-interacting GWAS variants for enhancer marks with respect to trait they belong. We chose a CVD-related trait (CAD) and compared with 5 variant size-matched non-CVD traits to demonstrate the specificity of such enrichments for the disease, and found that the enrichment for enhancer marks was significantly higher in CVD-related trait (Enrichment Comparison of CVD and Non-CVD Related Traits in the Data Supplement).

### Gene Enrichment Analysis of Target Genes of GWAS Variants for Discovery of Disease Associated Cellular Processes

We next asked if genes interacting with fragments carrying disease-associated variants are enriched for particular functions or pathways. To discover cell context–dependent signal, we performed a gene set enrichment analysis using genes interacting with GWAS variants themselves and those in LD for each cell type. We only included LD SNPs up to 80, 20, and 30 kb to the proxy SNP in AEC, ASMC, and mTHP-1–lipopolysaccharide cells. To assess the success of discovering novel processes or functions, we input the DEs containing these variants to Genomic Regions Enrichment of Annotations Tool software package to retrieve the gene sets independent of interaction information to perform the same enrichment analysis for comparison (Gene Enrichment Analysis Using topGO Package in the Data Supplement). We performed enrichment analysis separately for each cell and also combined to assess the specific contribution of each cell type. Comparison of the enriched terms by interacting or closest gene information (Genomic Regions Enrichment of Annotations Tool package) using a GO term semantic similarity measure revealed little overlap in between (Methods Gene Enrichment Analysis Using topGO Package and Table VIII in the Data Supplement). Figure 3D shows enriched biological processes when target genes from all cell types are merged. We located novel genes associated with known CVD complications, including response to lipopolysaccharide (GO:0032496, in AEC and mTHP1–lipopolysaccharide), phosphatidylinositol-3-kinase signaling (GO:0014065, in AEC), andSMAD protein signal transduction (GO:0060395, in AEC and ASMC). Moreover, we discovered genes and functions not previously associated with CVD onset and or progress, such as cilium assembly (GO:0060271, in AEC, 14 genes).

Ectopic deposition of calcium in arterial vessel walls leading to vascular calcification is a main feature of atherosclerosis and similar to the ossification process. Concordantly, terms, such as endochondral ossification, regulation of chondrocyte differentiation, regulation of osteoblast differentiation, positive regulation of ossification, were among the enriched functions. Eleven genes (BMP6, SMAD3, JAG1 [Jagged 1], PDLIM7 [PDZ And LIM Domain 7], SLC8A1, DLX5 [Distal-Less Homeobox 5], TEK, HOXA2 [Homeobox A2], EFEMP1 [EGF Containing Fibulin Extracellular Matrix Protein 1], RARB [Retinoic Acid Receptor Beta], and IL6 [Interleukin 6]) responsible for the above enrichments and only 4 (BMP6, TEK [TEK Receptor Tyrosine Kinase], SMAD3 [SMAD Family Member 3], and JAG1) interacted with lead SNPs, whereas the rest interacted with variants in LD with lead SNPs.

Fourteen target genes were involved in cilium assembly, including IFT74 (Intraflagellar Transport 74), a component of endothelial intraflagellar transport, which interacts with a CVD associated variant in the chr9p21 region (rs944797). It has been shown that endothelial cells can sense and respond to shear stress levels using their cilia and endothelial cilia were shown to deflect in response to blood flow rates. The deflection angle is regulated by calcium levels. Moreover, endothelial cilia inhibit onset of atherosclerosis in mouse models.

We identified several genes involved in leukocyte adhesion and vascular inflammation, key processes of atherosclerotic development. Examples of target genes include Cadherin 13 (CDH13 interacting with rs8055236) which has previously been shown to protect against atherosclerosis in experimental models, AMP-activated protein kinase (PRKAA2 [Protein Kinase AMP-Activated Catalytic Subunit Alpha 2] interacting with rs12239436) whose activity inhibits cell migration via phosphorylation of Pdlim5 (PDZ and LIM Domain 5) and BACH1 (BTB Domain And CNC Homolog 1; interacting with rs2832227), a transcriptional regulator which has been shown to be involved in atherosclerosis development in apoE deficient mice.

Other examples of plausible candidate genes for inflammatory cardiovascular disease include CD86 (CD86 Molecule; interacting with rs13083990), a receptor involved in the costimulatory signal essential for T-lymphocyte proliferation and interleukin-2 production and AKIRIN2 (interacting with rs6900057) a gene that has been shown to stimulate a proinflammatory gene in macrophages during innate immune responses.

### Expression Levels of Interacting Promoters Sharing Enhancers Are Correlated

Sometimes variants are contained within enhancers controlling multiple genes, suggesting such gene sets to be group- and pairwise coexpressed. Using data from the ASAP-Heart study (The Advanced Study of Aortic Pathology, heart tissue), we were able to test 75 AEC gene pairs sharing enhancers and could conclude that 34 (45.3%) are coexpressed at *P* value level 10^−3^; 18 of 75 (24.0%) also at *P* value level 10^−10^, and there is a strong enrichment over random background of gene pairs where only 23% are coexpressed (*P*=9.6e-06 by χ^2^ test). Excluding a large cluster of genes all interacted on by the same variant rs13083990, these percentages rises to 64.1% (p<10^−3^) and 38.4% (*P*<10^−10^), respectively.

**Figure 4.**
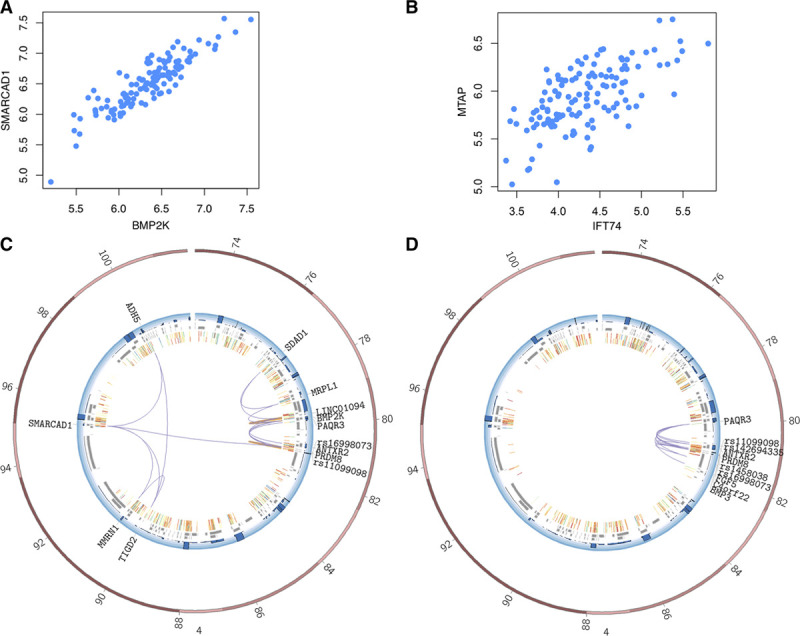
**Expression levels of promoters interacting with the same variant were correlated**. Expression correlation between (A) genes MTAP and IFT74 (P value 4.2×10−16) and (B) genes SMARCAD1 and BMP2K (P value 2.1×10−16) using aortic intima-media expression from 131 individuals. The values on axes are RPKM values, and each dot corresponds to each individual where both expression information are obtained from. **C** and **D**, Circos plot representation of interactions between rs16998073 and rest of the genome in **C** aortic endothelial cell (AEC) and **D** aortic smooth muscle cell for comparison. The plot spans chr4:73000000-103000000. The blue track represents gene expression levels, gray boxes represent transcripts, and innermost layers represent H3K27Ac marks in AEC cells (2 replicates). Purple arcs represent genome-wide association studies-promotor (Prom) or Prom-Prom, and orange arcs represent Prom-Distal interactions. BMP2K indicates BMP2 Inducible Kinase; BMP6, Bone Morphogenetic Protein; IFT74, Intraflagellar Transport 74; 6 MTAP, Methylthioadenosine Phosphorylase; RPKM, read counts per kilobase million; and SMARCAD1, SWI/SNF-Related, Matrix-Associated Actin-Dependent Regulator Of Chromatin, Subfamily A, Containing DEAD/H Box 1.

In Figure 4A, this is exemplified with coexpression plots for the *MTAP* and *IFT74* genes; both interacted on by the chr9p21 locus variant rs944797 in AEC. The genomic distance between the 2 gene promoters is in excess of 5 million bases. Even more extreme is the coexpression of genes *SMARCAD1* and *BMP2K* both located on chromosome 4 >15 Mb apart in AEC (Figure 4B). Both genes also interact a GWAS variant associated with diastolic blood pressure (rs16998073, *P* value=10^−21^), which itself interacts 2 other genes (*LINC01094* [Long Intergenic Non-Protein Coding RNA 1094] and *PAQR3* [Progestin And AdipoQ Receptor Family Member 3]) and 27 DEs (11 overlapping with H3K27ac marks; Figure 4C). Most of these interactions were specific to AEC (Figure 4D). LINC01094 is a noncoding RNA, and its expression levels are correlated with serum albumin (*P* value=10^−9^) and coexpressed with BMP2K (*P* value=1.26×10^−93^). Serum albumin levels are positively correlated with blood pressure. Moreover, PAQR3 levels modulate leptin signaling in mouse models, and leptin is found to mediate the increase in blood pressure associated with obesity.

## Discussion

Our aim in this study was to evaluate the contribution of high-resolution promoter-anchored regulatory interaction maps to locate the target genes of noncoding GWAS variants associated with vascular diseases. Although many GWAS variants are merely tags for the functional SNP within the same haplotype, some may still be or be close to the functional variant as suggested by their enrichment for enhancer marks and resequencing studies. Here, we show that targeting GWAS variants in capture Hi-C experiments can be a useful strategy in conquest for target gene associations because of lesser need for sequencing. We also uncover several enhancers regulating multiple genes and a strong correlation signal between such sharing enhancers, implying the underlying complexity of regulatory networks. A recent study showed the implicit wiring of enhancer redundancy in regulatory networks, where the system can tolerate loss of enhancers by connecting promoters to multiple enhancers.^47^ However, the case when multiple genes connected to the same enhancer could negatively affect the resilience of the network in the case of enhancer malfunction, potentially disturbing the coregulation of multiple genes.

We tackle the difficulty of locating the functional variant using LD information. When a GWAS SNP is associated with a trait, essentially any other variant on the same haplotype could be responsible for the association. However, because of sheer number of variants in LD, it is not straightforward to locate the functional one. The resolution in this study was around 750 bases, which allowed us to locate DEs containing variants in LD with CVD GWAS variants. Extending the genomic window around the DEs to 250 kb, we found that, it is possible to discriminate between functional and tagged variants using a double randomization procedure. DEs containing variants in LD with CVD GWAS variants showed better enrichment for histone enhancer marks and TF binding sites. We found that HiCap-identified loci are strongly enriched for genes identified by expression quantitative trait loci–based investigations of GWAS hits. Although the 2 methods are conceptually different, this overlap supports the idea that a diverse set of methods for functional genetics is advantageous when identifying causal genes from GWAS disease loci.

We confirm that it is only one-third of the time the enhancer is connected to the promoters of its nearest gene. We take on the challenge of assigning the correct genes to GWAS variants using promoter- and variant-anchored regulatory maps produced in 3 cell types. Indeed, we discover multiple biological processes and cellular structures that are associated with vascular disease pathology not by genomic but by functional studies. We were able to suggest variants that could be responsible for the perturbations of such processes or structures. Here, it is important to note that by only mining the variants associated with vascular disease traits, we will be able to discover the genes that are perturbed in a given pathway, process or structure. Discovery of the full network of genes within such processes or structure is beyond the scope of this study.

In summary, we provide high-resolution promoter-anchored regulatory networks of three cell types and list novel genes, processes, and cellular structures relevant for vascular disease pathologies, in particular, coronary artery and aortic diseases. We hope that the data and the methodologies in this study will aid us in our mission to further understand the contribution of noncoding genomic variation to complex disease biology.

## Sources of Funding

This work has been supported by the Swedish Research Council (Grant Agreement No. 78081).

## Disclosures

The computations and data handling were enabled by resources provided by the Swedish National Infrastructure for Computing (SNIC) through Uppsala Multidisciplinary Center for Advanced Computational Science (UPPMAX) partially funded by the Swedish Research Council through grant agreement no. 2018-05973.

## Supplementary Material

## References

[1] Roth GA (2017). Global, regional, and national burden of cardiovascular diseases for 10 causes, 1990 to 2015.. J Am Coll Cardiol.

[2] Reiner Z, European Association for Cardiovascular Prevention & Rehabilitation (2011). ESC/EAS Guidelines for the management of dyslipidaemias: the task force for the management of dyslipidaemias of the European Society of Cardiology (ESC) and the European Atherosclerosis Society (EAS).. Eur Heart J.

[3] Perk J, European Association for Cardiovascular Prevention & Rehabilitation (EACPR) (2012). European guidelines on cardiovascular disease prevention in clinical practice (version 2012): the fifth joint task force of the European society of cardiology and other societies on cardiovascular disease prevention in clinical practice (constituted by representatives of nine societies and by invited experts).. Int J Behav Med.

[4] Wang TJ (2011). Assessing the role of circulating, genetic, and imaging biomarkers in cardiovascular risk prediction.. Circulation.

[5] Duprez DA (2008). Identifying early cardiovascular disease to target candidates for treatment.. J Clin Hypertens (Greenwich).

[6] Chaturvedi N (2003). Ethnic differences in cardiovascular disease.. Heart.

[7] Zdravkovic S (2002). Heritability of death from coronary heart disease: a 36-year follow-up of 20 966 Swedish twins.. J Intern Med.

[8] Erdmann J (2018). A decade of genome-wide association studies for coronary artery disease: the challenges ahead.. Cardiovasc Res.

[9] Deloukas P, CARDIoGRAMplusC4D Consortium (2013). Large-scale association analysis identifies new risk loci for coronary artery disease.. Nat Genet.

[10] Malik R, AFGen Consortium; Cohorts for Heart and Aging Research in Genomic Epidemiology (CHARGE) Consortium; International Genomics of Blood Pressure (iGEN-BP) Consortium; INVENT Consortium; STARNET; BioBank Japan Cooperative Hospital Group; COMPASS Consortium; EPIC-CVD Consortium; EPIC-InterAct Consortium; International Stroke Genetics Consortium (ISGC); METASTROKE Consortium; Neurology Working Group of the CHARGE Consortium; NINDS Stroke Genetics Network (SiGN); UK Young Lacunar DNA Study; MEGASTROKE Consortium; MEGASTROKE Consortium: (2018). Multiancestry genome-wide association study of 520,000 subjects identifies 32 loci associated with stroke and stroke subtypes.. Nat Genet.

[11] Nelson CP, EPIC-CVD Consortium; CARDIoGRAMplusC4D; UK Biobank CardioMetabolic Consortium CHD working group (2017). Association analyses based on false discovery rate implicate new loci for coronary artery disease.. Nat Genet.

[12] Samani NJ, WTCCC and the Cardiogenics Consortium (2007). Genomewide association analysis of coronary artery disease.. N Engl J Med.

[13] Helgadottir A (2007). A common variant on chromosome 9p21 affects the risk of myocardial infarction.. Science.

[14] Reilly MP, Myocardial Infarction Genetics Consortium; Wellcome Trust Case Control Consortium (2011). Identification of ADAMTS7 as a novel locus for coronary atherosclerosis and association of ABO with myocardial infarction in the presence of coronary atherosclerosis: two genome-wide association studies.. Lancet.

[15] Horne BD (2008). Association of variation in the chromosome 9p21 locus with myocardial infarction versus chronic coronary artery disease.. Circ Cardiovasc Genet.

[16] Almontashiri NA (2015). 9p21.3 coronary artery disease risk variants disrupt TEAD transcription factor-dependent transforming growth factor β regulation of p16 expression in human aortic smooth muscle cells.. Circulation.

[17] Ghosh S (2015). Systems genetics analysis of genome-wide association study reveals novel associations between key biological processes and coronary artery disease.. Arterioscler Thromb Vasc Biol.

[18] Kessler T (2016). The impact of genome-wide association studies on the pathophysiology and therapy of cardiovascular disease.. EMBO Mol Med.

[19] Khera AV (2017). Genetics of coronary artery disease: discovery, biology and clinical translation.. Nat Rev Genet.

[20] Prashar Y (2017). Emerging role of various signaling pathways in the pathogenesis and therapeutics of atherosclerosis.. Rev Cardiovasc Med.

[21] Balding DJ (2006). A tutorial on statistical methods for population association studies.. Nat Rev Genet.

[22] Visscher PM (2017). 10 years of GWAS discovery: biology, function, and translation.. Am J Hum Genet.

[23] Dostie J (2006). Chromosome Conformation Capture Carbon Copy (5C): a massively parallel solution for mapping interactions between genomic elements.. Genome Res.

[24] Gusev A (2016). Integrative approaches for large-scale transcriptome-wide association studies.. Nat Genet.

[25] Cantor RM (2010). Prioritizing GWAS results: a review of statistical methods and recommendations for their application.. Am J Hum Genet.

[26] Chung D (2014). GPA: a statistical approach to prioritizing GWAS results by integrating pleiotropy and annotation.. PLoS Genet.

[27] Morris JA (2016). Using epigenomic data to inform genome-wide association studies of bone mineral density.. Ann Transl Med.

[28] Hauberg ME, CommonMind Consortium (2017). Large-scale identification of common trait and disease variants affecting gene expression.. Am J Hum Genet.

[29] Ma C (2018). The integrated landscape of causal genes and pathways in schizophrenia.. Transl Psychiatry.

[30] Zhu Z (2016). Integration of summary data from GWAS and eQTL studies predicts complex trait gene targets.. Nat Genet.

[31] Jia P (2014). Network.assisted analysis to prioritize GWAS results: principles, methods and perspectives.. Hum Genet.

[32] Leiserson MD (2013). Network analysis of GWAS data.. Curr Opin Genet Dev.

[33] Subramanian A (2005). Gene set enrichment analysis: a knowledge-based approach for interpreting genome-wide expression profiles.. Proc Natl Acad Sci U S A.

[34] Torkamani A (2008). Pathway analysis of seven common diseases assessed by genome-wide association.. Genomics.

[35] Yang J (2011). Genome partitioning of genetic variation for complex traits using common SNPs.. Nat Genet.

[36] de Lange KM (2015). Understanding inflammatory bowel disease via immunogenetics.. J Autoimmun.

[37] Baranzini SE, GeneMSA Consortium (2009). Pathway and network-based analysis of genome-wide association studies in multiple sclerosis.. Hum Mol Genet.

[38] Askland K (2009). Pathways-based analyses of whole-genome association study data in bipolar disorder reveal genes mediating ion channel activity and synaptic neurotransmission.. Hum Genet.

[39] Wang W (2017). Pathway-based discovery of genetic interactions in breast cancer.. PLoS Genet.

[40] Calabrese GM (2017). Integrating GWAS and co-expression network data identifies bone mineral density genes SPTBN1 and MARK3 and an osteoblast functional module.. Cell Syst.

[41] Gustafsson M (2015). A validated gene regulatory network and GWAS identifies early regulators of T cell-associated diseases.. Sci Transl Med.

[42] Huan T, International Consortium for Blood Pressure GWAS (ICBP) (2015). Integrative network analysis reveals molecular mechanisms of blood pressure regulation.. Mol Syst Biol.

[43] Sahlen P (2015). Genome-wide mapping of promoter-anchored interactions with close to single-enhancer resolution.. Genome Biol.

[44] Montefiori LE (2018). A promoter interaction map for cardiovascular disease genetics.. Elife.

[45] Anil A (2018). HiCapTools: A software suite for probe design and proximity detection for targeted chromosome conformation capture applications.. Bioinformatics.

[46] Hahne F (2016). Visualizing genomic data using gviz and bioconductor.. Methods Mol Biol.

[47] Osterwalder M (2018). Enhancer redundancy provides phenotypic robustness in mammalian development.. Nature.

