# Supplementary material for: High-Resolution Regulatory Maps Connect Vascular Risk Variants to Disease-Related Pathways
Source: Circ Genom Precis Med. 2019 Feb 20;12(3):e002353. doi: 10.1161/CIRCGEN.118.002353 (PMC8104016; doi:10.1161/CIRCGEN.118.002353)
Supplement: Supplementary file 1 [file hcg-12-e002353-s001.pdf]

Table of Contents

Materials and methods *2*

1. Cell Types 2
2. Chromosome conformation capture and targeted sequence capture 3
3. Chromatin Immunoprecipitation 4
4. RNA sequencing 4
5. Interaction Calling 4
6. Principal component analysis of the P-D interaction in relevant cell-types 5
7. Feature Enrichment Analysis 6
8. Interaction density comparison of promoter-GWAS hits 6
9. Double Randomisation for LD SNP Overlap 7
10. Enrichment comparison of CVD and non-CVD related traits 7
11. Gene Enrichment Analysis using topGO package 8
12. eQTL-enrichment of HiCap regions 8
13. Supplementary Figures 9

Supplementary Tables *18*

Materials and methods

1. Cell Types

Human aortic endothelial cells (AEC) were isolated from ascending aorta in patients undergoing elective open-heart surgery at the Cardiothoracic Surgery Unit, Karolinska University Hospital, Stockholm, Sweden. None of the patients had significant coronary artery disease, based on coronary angiography, and patients with Marfan syndrome were excluded (The ASAP/DAVAACA study). The study was approved by the Human Research Ethics Committee at Karolinska Institutet (application number 2006/784-31/1 and 2012/1633-31/4), Stockholm, Sweden; written informed consent was obtained from all the patients according to the Declaration of Helsinki, and methods were carried out in accordance with relevant guidelines. Following collection from operating theatre, biopsies were washed in PBS containing Calcium chloride and Magnesium chloride. Media and adventitia layers were then separated using forceps and tissues were laid in a non-tissue culture-treated Petri dish and subjected to enzymatic digestion using a solution of 1mg/mL of collagenase A (11088793001, Roche) in dispase 1U/mL (07923, Stemcell Technologies) for 20 to 25 min at 37°C, with regular gentle rocking of the dish. After incubation, endothelial side of the tissue was carefully scraped 5 to 7 times using a sterile scalpel. The collagenase solution containing endothelial cells was collected in a tube and the tissue was rinsed a couple of times with PBS and collected in the same tube. The solution was then strained using a 100µm cell strainer and centrifuged at 400g for 5 min. Finally, pellet was resuspended in 2.5mL of EBM-2 basal medium supplemented with EGM-2 BulletKit (CC-3162, Lonza) and dispensed in a 12.5cm2 flask previously coated with 0.2% bovine gelatin type B (G1393, Sigma). The next day, cells were gently washed twice with PBS and fresh endothelial cell growth medium was added. Hereafter, medium was replaced every 2 to 3 days. Upon confluence, cells were transferred to a 75cm2 flask and frozen at subconfluency in a solution of 90% FBS + 10% DMSO or used no later than P4.

Human aortic smooth muscle cells (ASMC) were purchased from Lonza (CC-2571) and cultured in SmBM basal media supplemented with SmGM-2 BulletKit (Lonza). ASMC were kept subconfluent and not used beyond passage 10. Constant environmental conditions of 37°C with 5% CO2 were kept throughout the growing of the cells.

For differentiation of THP-1 monocytes into macrophages a protocol using conditioned media as previously described by Whatling et al was used [1]. Briefly, THP-1 cells were grown in controlled environmental conditions in 37°C with a constant 5% CO2 supply. The base growing media for cells was RPMI 1640 completed with 10% of Fetal Bovine Serum (inactive), 1% of sodium pyruvate, 100 units/mL of penicillin and 100 µg/mL of streptomycin. THP-1 monocytes were split 1/5 after reaching a million cells per milliliter concentration and subsequently grown until the cell count reached 600,000 cells/mL. At this point, monocytes were differentiated to macrophages with the addition of phorbol 12-myristate 13-acetate (PMA, 1mg/mL) to the final concentration of 50 ng/mL. After 24h, half of the differentiated THP-1 macrophages in growing flasks were cross-linked with 1% formaldehyde solution (after 2 hours) and another half was stimulated with 1 µg/µL of lipopolysaccharides from *Escherichia coli* O55:B5 (Sigma-Aldrich, L6529) for 2h prior to cross-linking.

2. Chromosome conformation capture and targeted sequence capture

The cells for the high-throughput chromosome conformation capture followed by targeted sequence capture (HiCap) were fixed in 1% formaldehyde solution for 10 minutes and the reaction was quenched with an excess of glycine at final concentration of 0.125M. The cells were harvested in a cold environment in a presence of protease inhibitors. After pelleting via centrifugation, the cells were lysed and nuclei were isolated. Sodium dodecyl sulfate (SDS) was used to solubilize the chromatin to aid the molecular accessibility for subsequent 4-cutter enzymatic digestion with 1μL/μg of FastDigest *Mbo*I (↓GATC; Thermo Fisher Scientific) for 4.5 hours at 37°C. Before the digestion, SDS was quenched using surfactant Triton-X to avoid any undesired impact on enzymatic activity. Protruding 5' DNA strand ends left by restriction enzyme were filled using Klenow fragment of DNA Polymerase I with biotin-14-dATP presence in the environment. This action was taken for later avidin-based selection of target ligation reaction product while removing reaction artifacts. The enzymatic activities of Klenow fragment were quenched by a brief sample incubation at 75°C in a presence of 10mM of EDTA. The resulting material of chromatin enzymatic digestion with biotin labeled blunt DNA ends was then subjected to proximity ligation reaction with 12 Weiss units of T4 DNA ligase (New England Biolabs) for 4.5 hours at 16°C. The reaction design was set to favor intra-molecular ligation in each chromatin complex. After ligating the DNA that was spatially close-by during the cell fixing, the formaldehyde crosslinks were thermally removed at 65°C for 8 hours with the aid of Proteinase K in reaction environment. The resulting chimeric DNA complexes were further purified with phenol-chloroform-isoamyl alcohol (25:24:1 (v/v/v), pH 8.0) and precipitated in absolute ethanol saturated with sodium acetate pH 5.2. Any RNA contamination was removed by RNase A treatment of the samples for 1 hour at 37°C. At this point, controlling the quality and quantity of the DNA assessed chromosome conformation capture complexes and control reactions taken at different time-points of the experiment. All quality and quantity controls throughout the protocol were performed by Qubit fluorometric quantitation (Invitrogen) and 2100 Bioanalyzer system (Agilent).

Un-ligated ends containing biotin were removed by utilizing a strong 3'-5' exonuclease activity of T4 DNA Polymerase for 15 minutes at 12°C. Subsequently the chimeric DNA was fragmented into 100-200 bp fragments using sonication system by Covaris Inc. The ultrasound shearing of the DNA was performed by the following protocol: 6 cycles of 60 seconds with 10% duty cycle, intensity of 5, and cycles per burst of 200. The fragments were then used to prepare DNA sequencing libraries by employing KAPA HTP Library Preparation kit for Illumina Platforms. The ends of the fragments were repaired and the poly-A "tail" was added to facilitate later Illumina TruSeq LT adapters. The manufacturer's protocol was then modified to incorporate avidin-biotin selection of target DNA fragments by using MyOne C1 Streptavidin beads. The following experimental step of Illumina TruSeq adapter ligation was performed while DNA fragments were bound to aforementioned streptavidin magnetic beads. The beads were then washed and put into suspension with water to perform amplification of the prepared sequencing libraries. Thermal cycling was done by initial denaturation of the DNA for 45 seconds at 98°C followed by 6 cycles of denaturation of 15 seconds at 98°C, primer annealing of 30 seconds at 60°C, strand extension of 30 seconds at 72°C, and finalized with a single final elongation of 1 minute at 72°C.

The obtained library pool was later used for enrichment by custom target capture using parts of SureSelect XT Target Enrichment System for Illumina Paired-End Multiplexed Sequencing libraries (Agilent). Illumina adapter sequences were first blocked by xGen Universal blocking oligonucleotides (Integrated DNA Technologies) by denaturing the libraries at 95°C and then melting the blocking nucleotides at 65°C. Custom pre-designed RNA probe panel was then used to hybridize to the libraries at stringent conditions for 24 hours as proposed by SureSelect system manufacturer. After the hybridization, the selected libraries were washed at stringent conditions to remove un-hybridized

DNA, followed by 8-cycle post-capture-PCR as described in manufacturer's protocol. The resulting enriched DNA libraries were purified and in-house sequenced via Illumina single index, paired end sequencing on NextSeq 500 platform (Illumina Inc).

3. Chromatin Immunoprecipitation

AEC and ASMC were cross-linked with 1% formaldehyde for 10 min. Around 5 million cells were used for each experiment and each experiment is performed in duplicates. All steps were performed at 4°C unless otherwise indicated. Cells were lysed in swelling buffer (100 mM Tris at pH 7.5, 10 mM KOAc, 15 mM MgOAc, 1% igepal, PIC) for 10 min followed by Dounce homogenization. Nuclei were pelleted at 4000 rpm for 5 min and lysed in modified RIPA buffer (PBS with 1% NP-40, 0.5% sodium deoxycholate, 0.1% SDS, 1 mM EDTA, PIC) for 10 min. Chromatin was sonicated to 150–300 bp using a Diagenode bioruptor. Insoluble material was removed by centrifugation at 13,000 rpm for 10 min followed by preclear with StaphA cells for 15 min. One microgram of primary antibody/5 million cells (Diagenode, C15410196-10) was incubated with precleared chromatin for 16 h. Anti-rabbit secondary antibodies (Millipore) were added for 1 h. Ten microliters of StaphA cells/5 million cells was added for 15 min at room temperature. StaphA cells were washed twice with dialysis buffer (50 mM Tris at pH 8, 2 mM EDTA, 0.2% sarkosyl) and four times with immunoprecipitation wash buffer (100 mM Tris at pH 8, 500 mM LiCl, 1% NP-40, 1% sodium deoxycholate). Chromatin was eluted off StaphA cells in 1% SDS and 50 mM NaHCO3. For re-ChIP, eluted chromatin was diluted 10-fold in modified RIPA without SDS, and second primary antibody was added overnight. For ChIP-seq and ChIP-qPCR, 200 mM NaCl was added, and cross-links were reversed for 16 h at 67°C. DNA was purified using PCR purification kit (Qiagen), eluting in 50 µL of water. One microliter of ChIP DNA was used for qPCR. For ChIP-seq, libraries were prepared using the Mondrian (NuGen) and size-selected using Pippin Prep (Sage Science). Libraries were sequenced with 1 × 50 base pair reads on the NextSeq 500 platform (Illumina Inc).

4. RNA sequencing

We performed RNA-seq on AEC and, ASMC and mTHP-1-LPS cells according to the following protocol, each experiment is performed in duplicates. RiboCop Kit (Lexogen) is used for RNA extraction, rRNA was depleted using 900 ng of each prep of total RNA according to manufacturer's protocol. Libraries from 8 uL of depleted RNA were prepared according to manufacturer's protocol ("paired end 100 bp" sequencing was used to set reagent concentrations and thermal cycling conditions). The libraries were sequenced 1x75 base pair reads on the NextSeq 500 platform (Illumina Inc).

5. Interaction Calling

HiCap provides p-values relative to the null-hypothesis that physical 3D-distance is proportional to genomic distance. Contact occurrences (abbreviated SP for Supporting Pairs) among genomic segment pairs of interest are related to a carefully selected set of negative controls at corresponding genomic distance. Negative controls are regions with no known regulatory activity and far from promoters at a set distance, which in this study was 50 kb [2]. Recent performance comparison [15] revealed that Hi-Cap outperforms other sequence capture based methods at genomic distances above 500 kb, and is competitive also below that [2].

Only interacting segments meeting a requirement of four SPs and a p-value below 10-3 were taken forward. Each HiCap analysis was replicated twice and an interaction was only considered if SP and p-value requirements were met for both.

For the HiCap experiments we fragment the genome using a 4-cutter restriction enzyme (DpnII); the average fragment length for DpnII is 650 bases for the human genome.

When we call promoter-anchored interactions from HiCap data, we use the DpnII-fragments to find promoter interacting regions, i.e. we call interactions on DpnII-restriction fragment level. Therefore, our average interacting fragment length is 749 bases, slightly longer than the expected length.

Using an empirically derived background distribution could reduce the number of false positives. Despite careful selection of negative controls not overlapping with promoter or enhancer sequences, there could still be a subset of regions which could be regulatory and involve in genomic interactions. This could then result rather miss interactions (false negative) rather than detecting more false positives since the signal from "negative controls" would be higher due to some negative controls involved in regulatory interactions. We tried to estimate the false discovery rate by comparing the number of interactions between promoters and negative controls normalised by their set size (Table 1). Such calculation shows us our false discovery rate estimate is around 9%.

$$\mathrm{FDR} = \frac{(\mathrm{P} * \mathrm{INC})/\mathrm{NC}}{\mathrm{IP}}$$

Cell Type	BAV	SMC	mTHP1-LPS
Number of Negative Controls (NC)	722	722	722
Number of Promoters (P)	13,472	13,472	13,472
Number of Interactions of Negative Controls (INC)	335	192	90
Number of Interaction of Promoters (IP)	69,753	38,759	19,920
False Discovery Rate (FDR)	0.089	0.09	0.084

Table 1. The number of expected interactions of promoters (P*INC)/NC is divided by the observed number of interactions (IP) to estimate the false positives.

6. Principal component analysis of the P-D interaction in relevant cell-types

HiCap experiments generated contact occurrences (SP) information was used to generate the supporting read pairs (CPM) values for individual probe anchored gene. The total SP read counts for probed genes were normalized by total reads pairs sequenced in the HiCap experiments to generate CPM values. Using these CPM metrics for individual probe anchored genes, we did PCA separation of expressed and unexpressed genes in all cell types.

We first identified 8,009 genes expressed in all cell types and associated targeted gene probes in HiCap experiments. Furthermore, we also identified 5921 unexpressed genes but associated targeted gene probes in all cell types. Using these expressed and unexpressed genes we selected distal interactions and associated CPM supporting pair for all relevant cell types. We then carried out principal component analysis on these distal regions normalized read count of expressed and unexpressed

probe genes. We want to show here that the interaction datasets can separate cell types independent of the gene expression information. To achieve this, we took genes that have no expression value (FPKM is equal to zero) in all cell types. We then calculated normalised read counts (CPM) for regions that non-expressed genes interact in each cell type, and performed a principal component analysis for the CPM values of the interacting regions and found that it is possible to separate the cell types based on the different interaction profiles of non-expressed genes common to all cells. Since these genes are common to all three cell types and have no gene expression information in none of the cell types, the separation comes from the different interaction profiles of these genes in each cell type.
Similar PCA separation was made for all expressed genes based on RNA FPKM dataset as shown in Figure 1 (a,c).

7. Feature Enrichment Analysis

Several ChIP-seq peak enrichment analyses were performed. Tissue relevant DNaseI, histone modification (H3K27ac and H3K4me1) and transcription binding datasets were downloaded from the ChipAtlas (chip-atlas.org); in cases were several ChipAtlas datasets met requirements these were merged before analysis. Overlap occurrences among HiCap features were compared with those for distance-from-promoters and length controlled random sequences. That is, a promoter, a promoter-distal element distance and a segment length were separately sampled and thus a negative control sequence obtained. Table 2 lists the percentage overlaps of ChIP-Seq datasets to distal regions.

Datasets	AEC Prom-Distal (69,573)	ASMC Prom-Distal (38,759)	mTHP-1-wLPS (19,920)
**Total Overlap**	**30,342 (43.5%)**	**14,515 (37.5%)**	**6,111 (30.7%)**
AEC (H3K27Ac)	17,767 (25.5%)	-	-
ASMC (H3K27Ac)	-	5,781 (14.9%)	-
HAEC TF	6,656 (9.5%)	-	-
Cardiovascular DNase	23,683 (34%)	11.986 (30.9%)	-
Cardiovascular TF	17,353 (24.9%)	8,336 (21.5%)	-
Macrophages DNase	-	-	991 (4.6%)
Macrophages Histone	-	-	2,661 (13.4%)
Macrophages TF	-	-	5,511 (27.7%)

Table 2. the percentages of overlap of ChIP-Seq datasets to distal regions.

8. Interaction density comparison of promoter-GWAS hits

Figure 2a aims to show that the detected GWAS-promoter interactions are not due to the fact that the GWAS variants were targeted. In order to investigate this, we look for interaction density on each side of the GWAS interacting promoter. The “hit side” refers to the side of the promoter the GWAS SNP lies and the opposite side is the other side of the promoter with the exact same distance as the GWAS SNP. The sketch diagram below shows an interaction between GWAS variant and promoter and denotes the “hit side” and “opposite side”. We then divide the space around the GWAS variant into bins and count for the **prom-distal** interactions of the GWAS interacting promoter in both hit and opposite side, since in the prom-distal set, it is only the promoter that is targeted. If we were to obtain GWAS-promoter interactions due to targeting GWAS variants, we would not see the pattern in Figure 2a, i.e. higher density of significant prom-distal interactions only around the GWAS variant (i.e. hit side). It is also important to note that we see the same pattern if we perform the same analysis using raw read pairs and/or smaller bins.

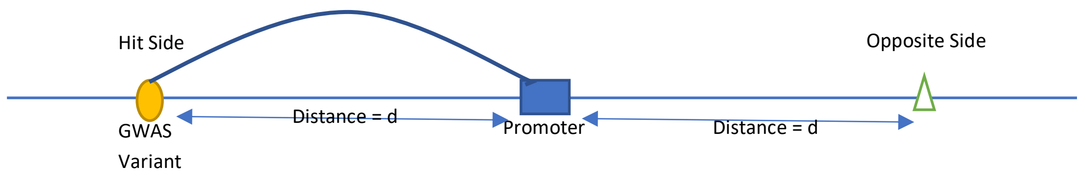

9. Double Randomisation for LD SNP Overlap

We used the SNPsnap tool (https://data.broadinstitute.org/mpg/snpsnap/) to obtain a set of SNPs that matched the CVD_GWAS set in terms of size, minor allele frequency, LD structure, gene density, distance to nearest gene using European 1000 Genomes Phase 3 population. These constituted the random SNP sets.

We then used SNiPA tool to extract variants in linkage disequilibrium to variants of interests (https://snipa.helmholtz-muenchen.de/snipa/index.php?task=proxy_search). We used Europan 1000 Genomes phase 3 population and LD threshold of 0.8 within 250 kb window of query variant. Using SNiPA, we generated the LD SNP set for CVD_GWAS and random SNP sets (20).

We also generated random interaction sets (100). We first fitted the length distribution of interactor set using log normal distribution to obtain the mu and sigma parameters. We then generated random coordinates matching the size and length distribution of the observed set using bedr package implemented in R. We then matched the fraction of interactors within genic regions to that of observed set using bedtools.

We overlapped both CVD_GWAS and random SNP sets and the corresponding LD SNP sets to both observed DE set and randomly generated DE sets. We assigned statistical significance to the signal obtained by overlapping to the observed DE set and CVD_GWAS LD set by comparing the overlaps of observed DE set to random SNP sets, the overlaps of random SNP sets to CVD_GWAS sets and the overlaps of random DE sets to random SNP sets. For all cell types, the overlap was statistically significant for only observed DE set to CVD_GWAS LD set (figure 3a, supp. figure 2a and b). All random sets and codes are available upon request. All CVD_GWAS SNPs or those in LD are reported in supplementary table 7 (Column “type” PD_LD denotes an interaction with an LD SNP).

10. Enrichment comparison of CVD and non-CVD related traits

We have chosen the following traits for comparison: Schizophrenia, Heel bone mineral density, Prostate cancer, Crohn's disease and Body Mass Index. None of the traits showed any enrichment towards histone or transcription factor binding site datasets in HAEC. Due to small overlap sizes (i.e. the number of variants overlapping with relevant histone mark or transcription factor), it was not possible to produce similar enrichment scores compared to matched negative control sets, and therefore could not represent them in the same manner as Figure 3a. However, the table 3 shows the overlap counts of for each trait with promoter-distal and ChIP-seq H3K27Ac dataset (the dataset used to produce Figure 3a). We have also taken the “coronary artery disease” trait for comparison: 33% of coronary artery disease promoter-interacting GWAS SNPs also overlapped with H3K27Ac mark in HAEC. Whereas the same number for other traits is significantly lower (p value < 1e-4 for all comparisons, Chi-squared test) (Table 3).

Trait	Total # of SNPs	Overlapped with prom-distal dataset	Overlapped with prom-distal and H3K27Ac dataset (HAEC)	Ratio

Schizophrenia	299	33	2	0.06
Heel bone mineral density	283	45	3	0.06
Prostate Cancer	267	21	2	0.1
Crohn's Disease	241	19	0	0
Body Mass Index	675	25	1	0.04
Coronary Artery Disease	442	46	15	0.33

Table 3. The ratio of GWAS-SNPs of various traits overlapping both with a distal region and regulatory element.

11. Gene Enrichment Analysis using topGO package

Next, we asked if there are any gene sets or pathways enriched within the target gene set of CVD_GWAS. We used topGO package implemented in R (http://bioconductor.org/packages/release/bioc/html/topGO.html). We have performed the enrichments using three different universal gene sets: GENCODE, RefSeq and custom gene set containing only those targeted by at least one probe in the experiments. All three gave very similar enrichment profiles, therefore decided to use the smallest (19,710 genes) gene set. The Fisher test and weight01 algorithms are used to calculate enriched GO terms.
We then performed the same enrichment analysis using only the closest genes to the SNPs. We used GREAT package implemented in R (rGreat) to locate the closest genes to SNPs in "basalPlusExt" mode.
To compare the enriched terms, we used GOSemSim package implemented in R (https://bioconductor.org/packages/release/bioc/manuals/GOSemSim/man/GOSemSim.pdf) to quantitatively measure the similarity between the GO terms. We used "Wang" similarity score and similarity threshold is set to 0.7.

12. eQTL-enrichment of HiCap regions

For each set of distal regions, a corresponding set of random regions that had similar length and similar gene distribution. In both the Hi-Cap and the random definition files, eQTL associations where calculated between all SNPs located in the source region and all genes located in target region. The eQTL-analysis was performed using the ASAP data set, as also described previously [3] as well as in above cell-extraction analysis. The ASAP/DAVAACA study was approved by the Human Research Ethics Committee at Karolinska Institutet (application numbers 2006/784-31/1, 2012/1633-31/4), Stockholm, Sweden. The primary analysis was performed in heart-tissue (n=127), and secondary analyses was performed in aorta intima-media (n=139) and adventitia (n=133) as indicated. Analysis was performed using the precise regions defined by the HiCap experiment (flanking region = 0), as well as with flanking regions of 1kb, 3kb and 5kb according to the hypothesis that LD-blocks may affect eQTL findings outside of the Hi-Cap regions. The eQTL calculations were performed using linear regression of log2 transformed gene expression as function of effect allele count. Comparison of enrichment was done using the qqplot function available in R 3.4.1.

Note that there was no P-value cut-off in the eQTL enrichment analysis in figure 3b. The strongest eQTL associations found between each promoter-distal block was plotted (Y-axis) against a size- and distance controlled random set (X-axis). That means that even though some of the eQTL associations were weak - at P-values up to 1, as seen in the lower-left of the figure, then overall there was a strong deviation towards more eQTL-association when using the prom-dist block with eQTL association signals significant at up to P=1e-8.

13. Supplementary Figures

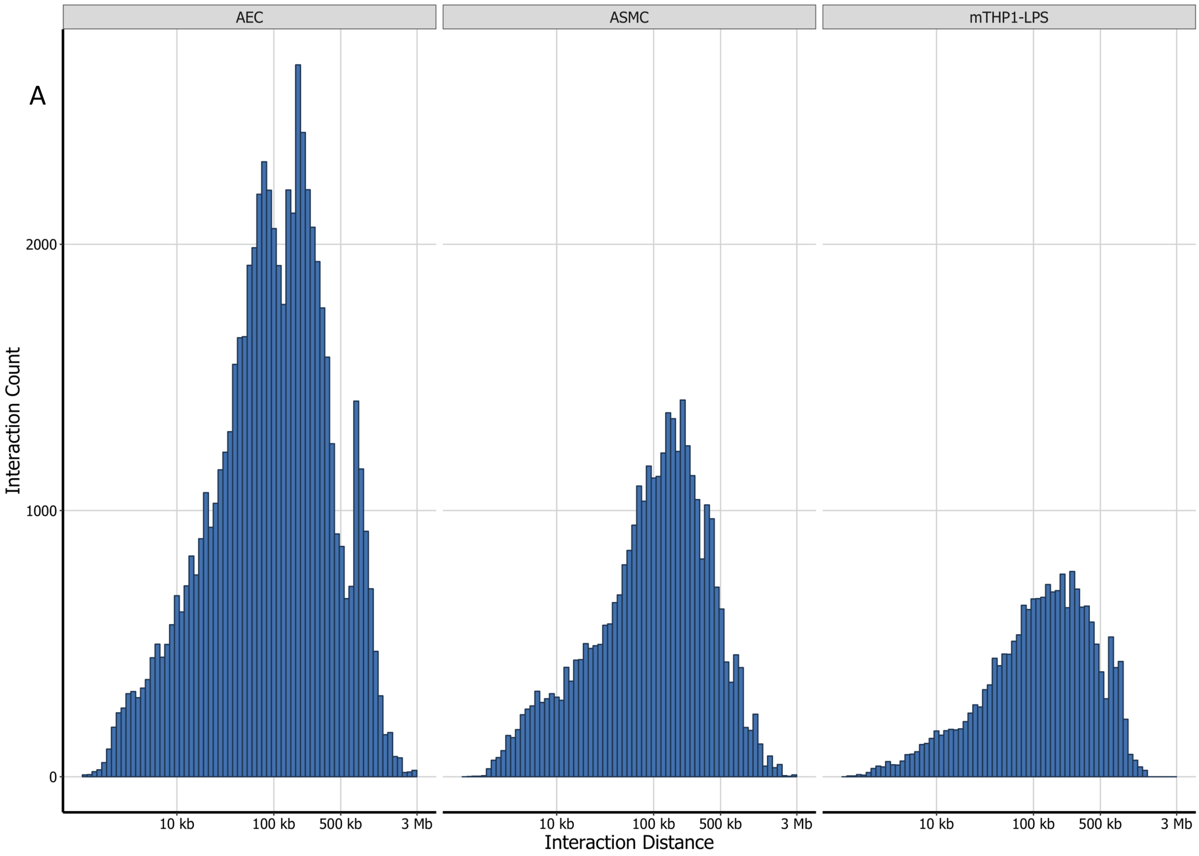

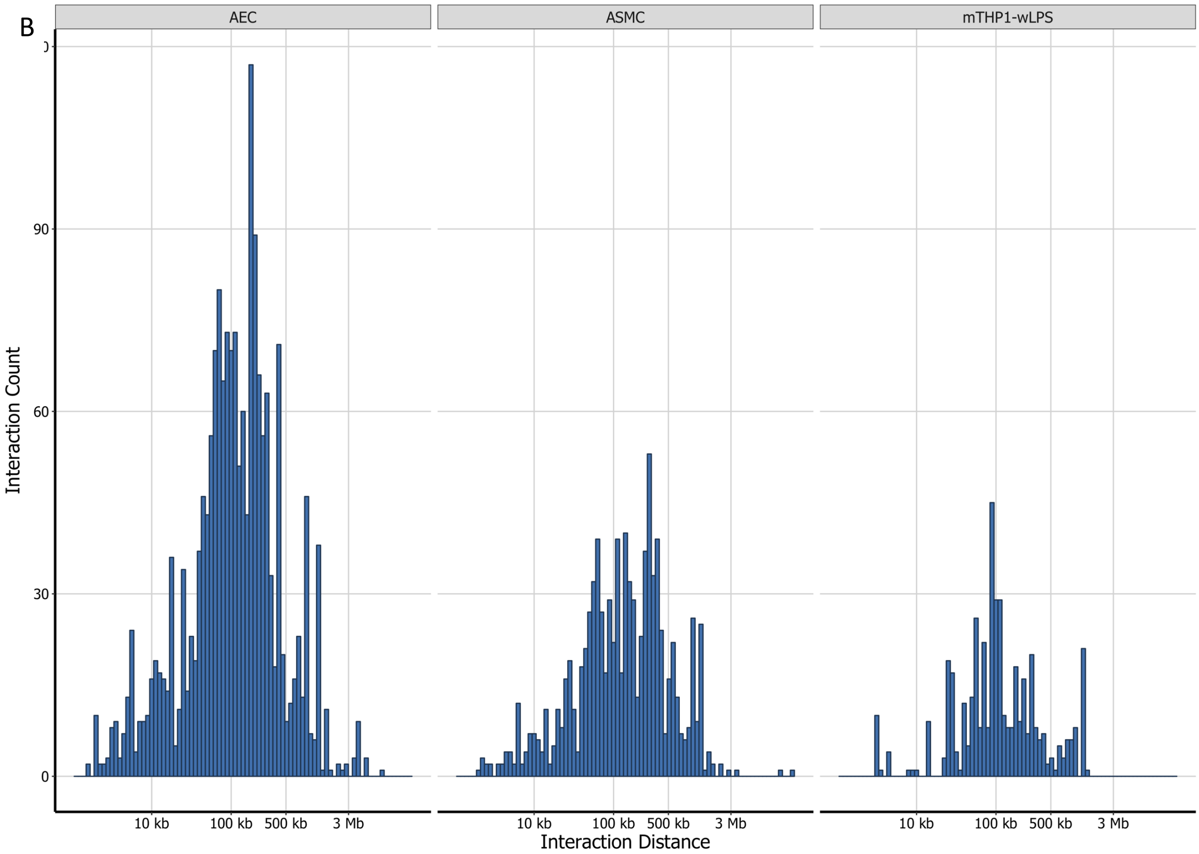

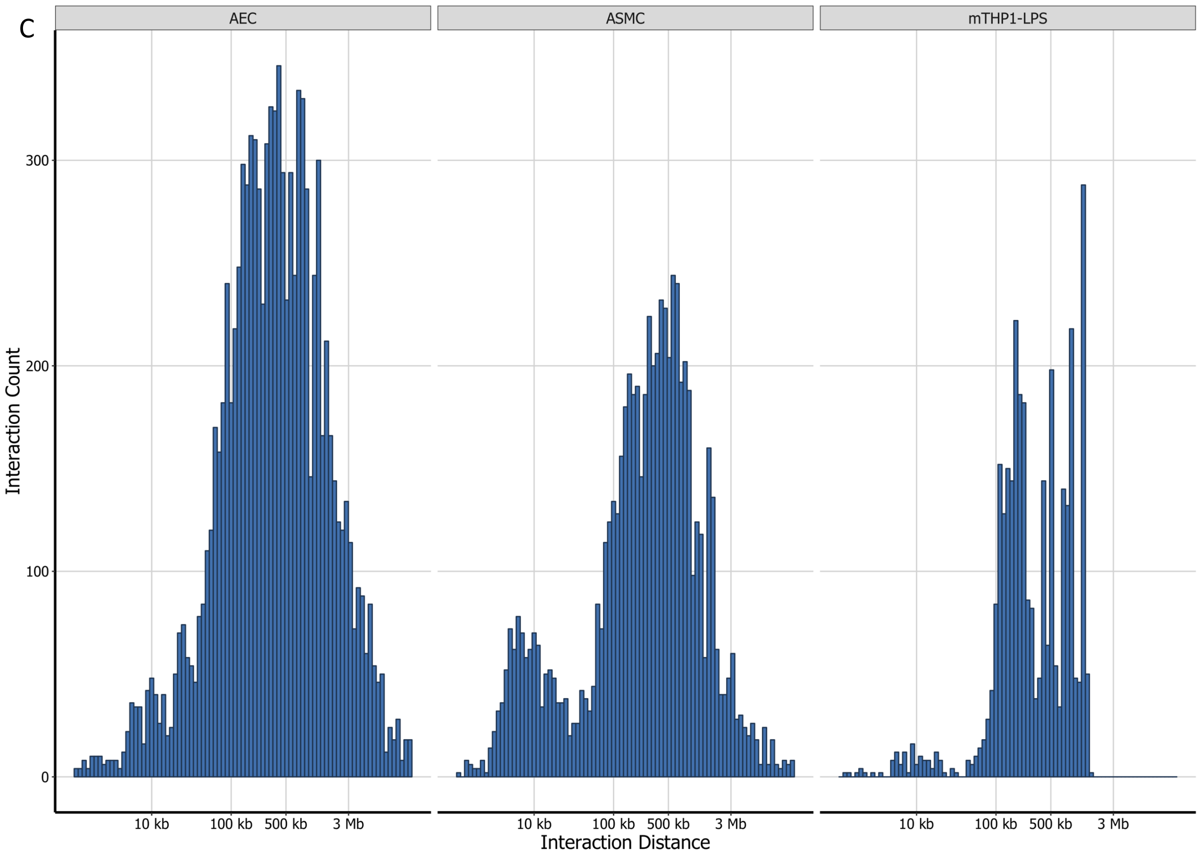

Supplementary Figure 1 Interaction distance counts with respect to interaction type, A) Promoter-Distal B) promoter – promoter and promoter - GWAS, in different cell type: a) AEC, b) ASMC and c) mTHP1.

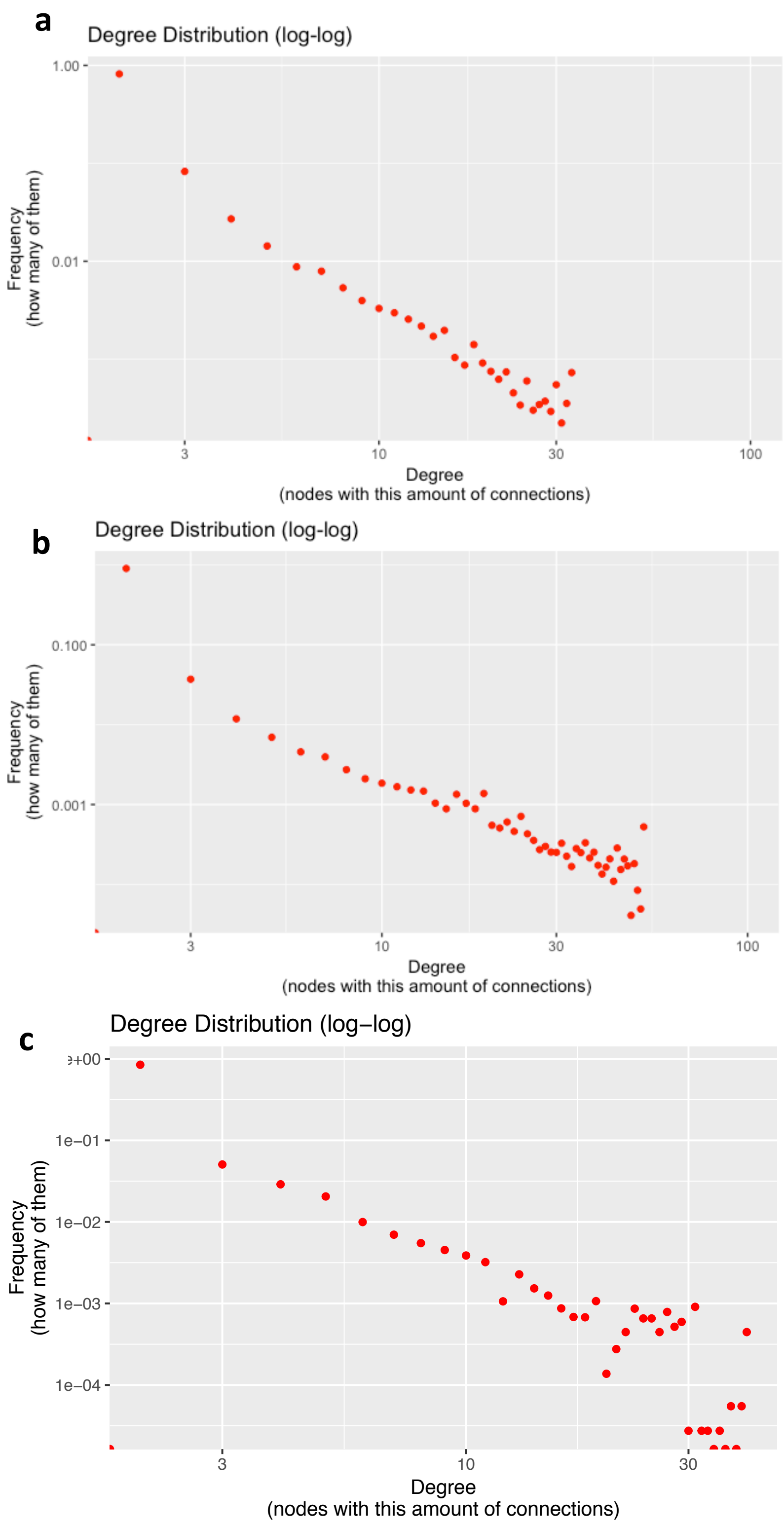

Supplementary Figure 2 Degree distribution of interacting promoters in all chromosomes in a) AEC, b) ASMC, and c) mTHP-1-LPS cells.

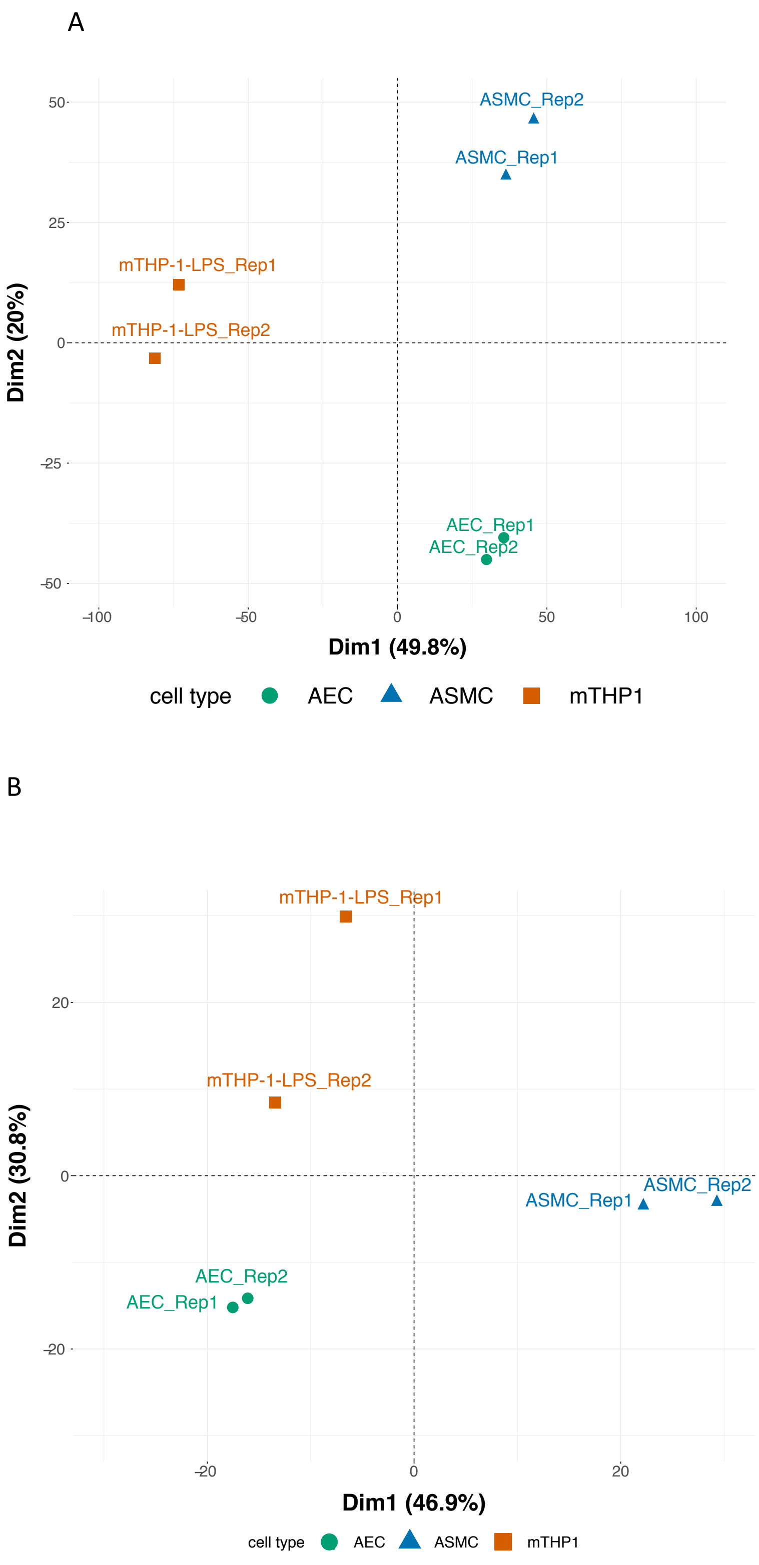

Supplementary Figure 3 PCA separation of expressed genes and interactome profiles of expressed genes in all cell types. a) PCA was performed in common expressed genes in all cell types based on RNA FPKM counts. The clustering of the replicates together indicates the distinct transcriptome profile of

individual cell types. b) Similar PCA separation was performed on interaction dataset of genes based on supporting pair FPKM counts. The corresponding PCA plot indicates that the interaction profiles of expressed genes were distinct as well

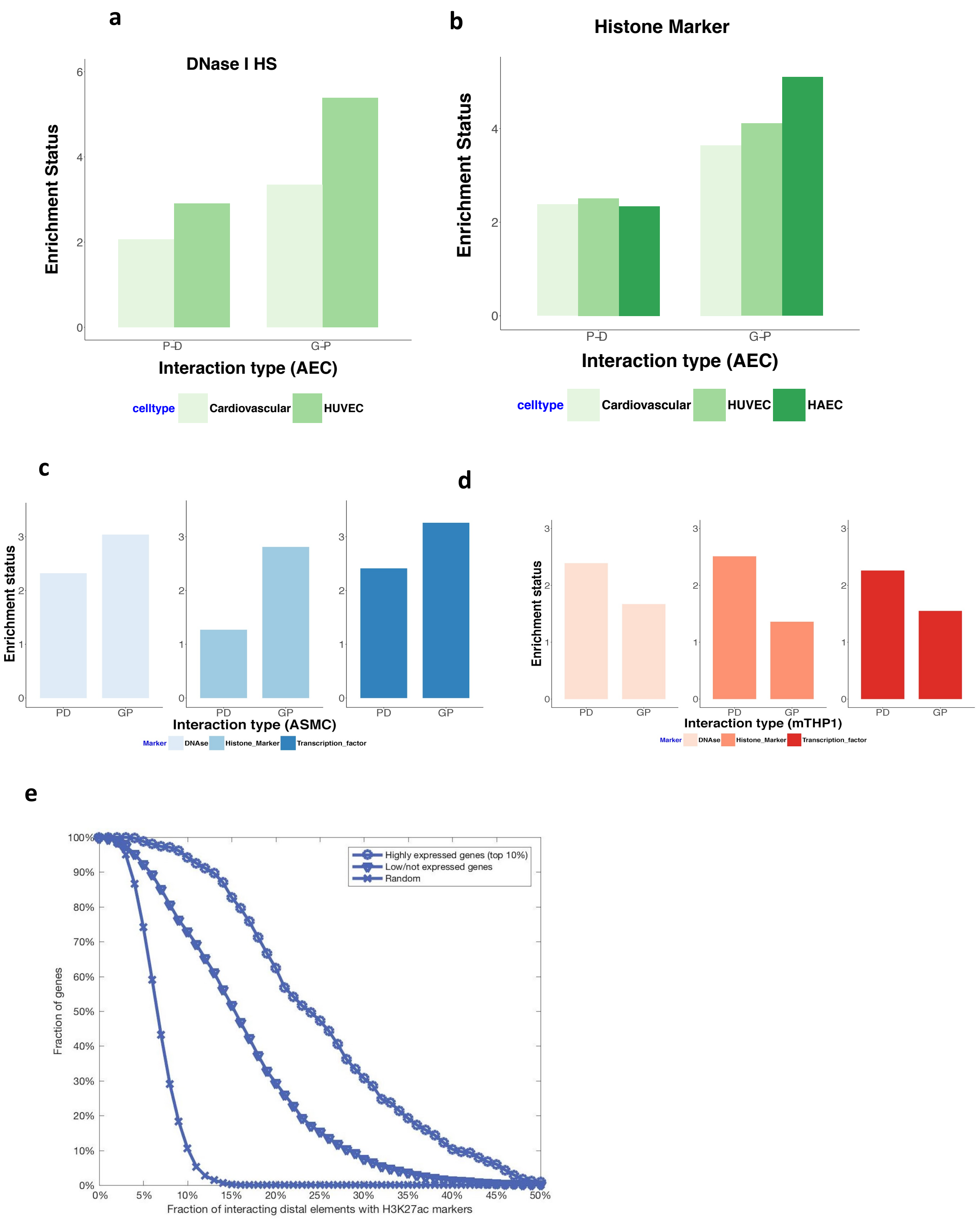

Supplementary Figure 4: Enrichment status of AEC, ASMC and mTHP1-LPS P-D and GP datasets with respect to regulatory markers from cardiovascular relevant cell types. Marker data are in all cases downloaded from ChipAtlas. All enrichment states are calculated with respect to a segment-length and distance-from-promoter controlled random set. (a,b) AEC P-D and GP interaction datasets were overlapped with regulatory DNaseI hypersensitive (HS) and histone marker genomic regions from Cardiovascular, HUVEC and HAEC cell types. Both P-D and GP interaction showed increased enrichment

when compared to length and distance compared random sets. (c) Similar enrichment analysis was performed in ASMC P-D and GP interaction dataset with respect to regulatory DNaseI HS, histone marker and transcription factor genomic regions from human cardiac myocytes (HCM) and coronary artery (CA) smooth muscle cells. (d) Enrichment analysis of mTHP1 P-D and GP interaction dataset overlapped with regulatory DNaseI HS, histone marker and transcription factor genomic regions from macrophages and THP1. (e) AEC genes were organised in decreasing order by the fraction of interacting distal elements which overlap cardiovascular H3K27ac markers. Interactors to the 10% most highly expressed genes were compared to interactors for remaining genes and to a set of length and distance controlled random regions. P-value for difference between gene sets is about 4.6*10-78.

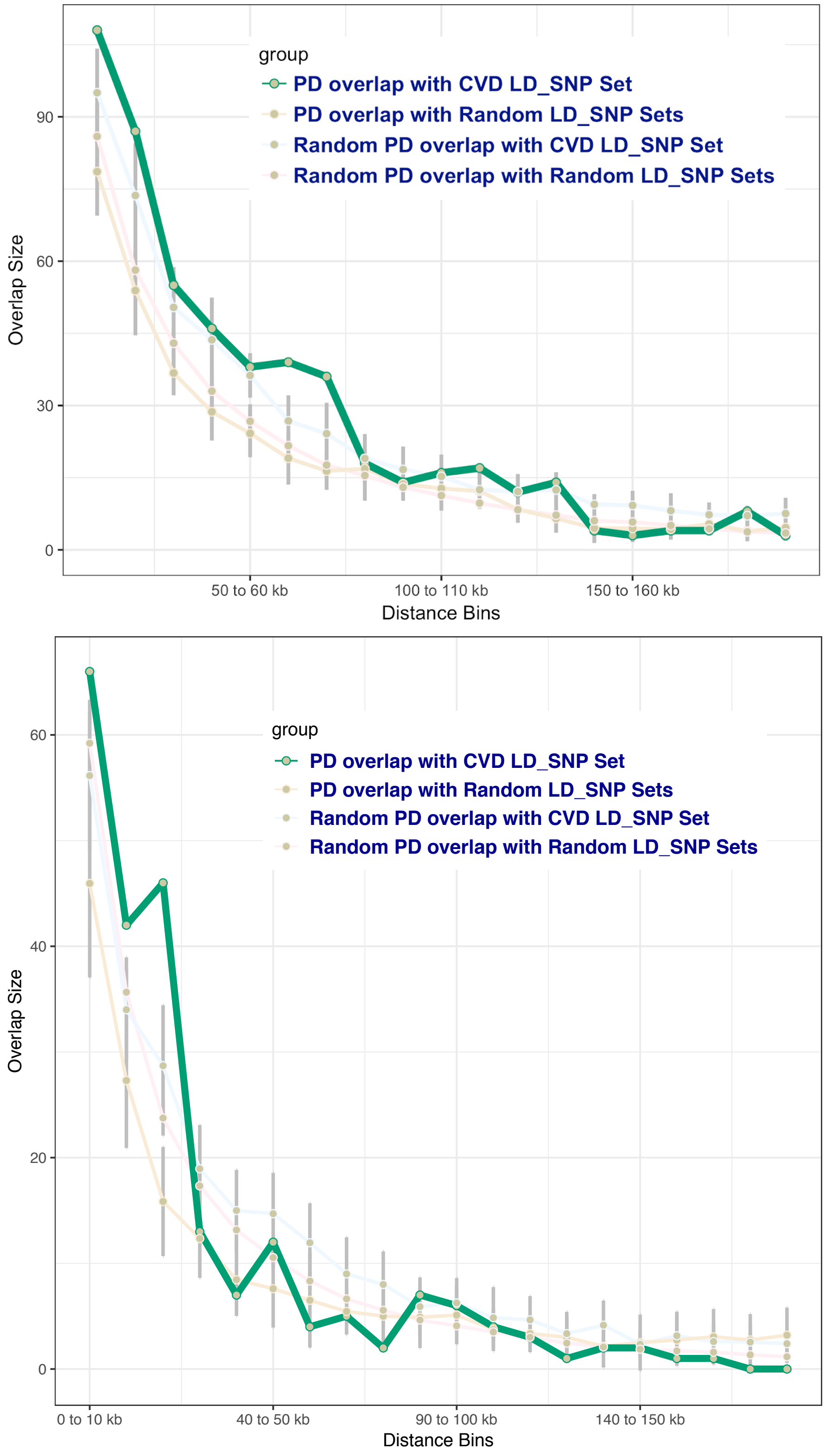

Supplementary figure 5. a) Enrichment of P-D set for SNPs in LD with CVD_GWAS set in ASMCs. Only the first two bins (10 and 20 kb) and 60 and 70 kb bins showed enrichment. b) Enrichment of P-D set for SNPs in LD with CVD_GWAS set in mTHP1-wLPS. Only first three bins (10, 20 and 30 kb) showed enrichments.

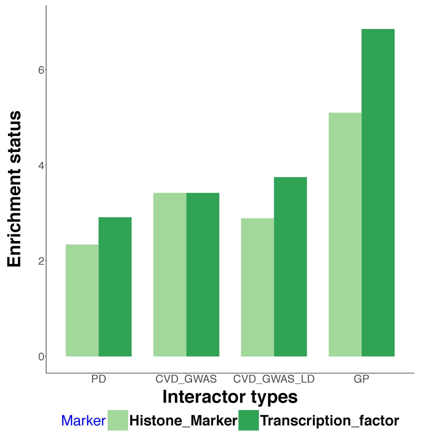

Supplementary Figure 6. Overall enrichment of the Prom-Dist (AEC) datasets to the functionally annotated transcription factor and histone markers from Human Aortic Endothelial cell (HAEC) chip-atlas database. The enrichment status was assigned with respect to segment-length and distance-from-promoter controlled random set datasets.

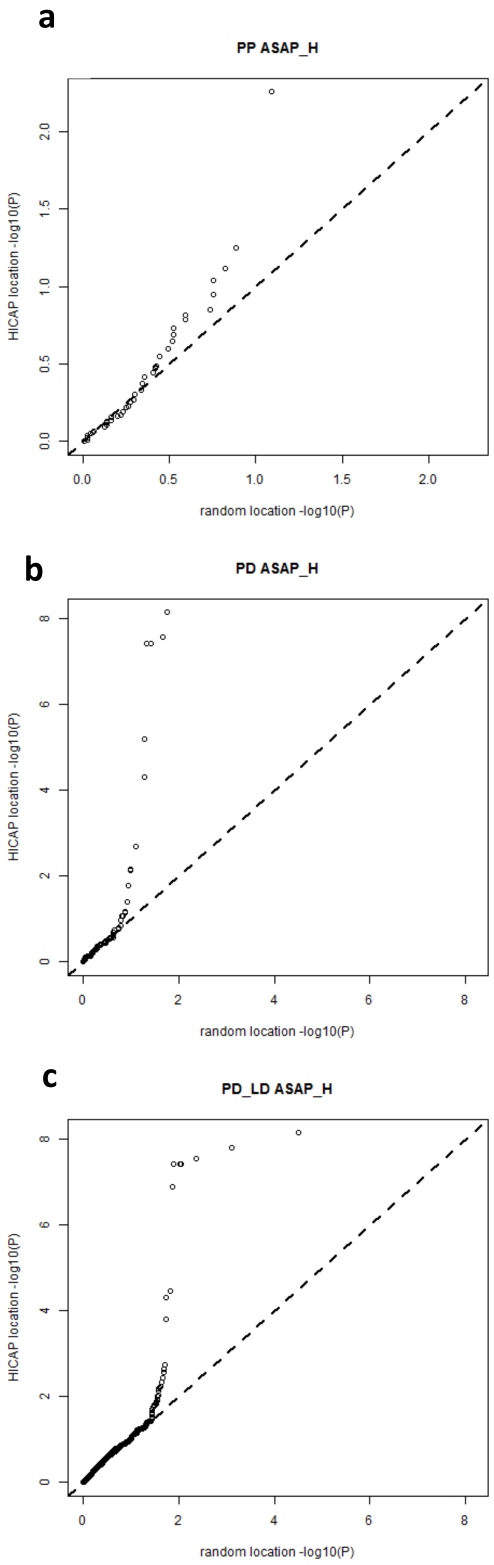

Supplementary figure 7. eQTLs contained in, respectively (a) the G-P set, (b) a selected subset of the P-D set, and (c) hits in linkage disequilibrium (LD) with the P-D set. Deviation from diagonal is present in all three cases.

Supplementary Tables

Supplementary Table 1. Coordinates of the sequence capture probes used in this study. Each entry is 120 bases long corresponding to the feature. There are 12 worksheets in this table, grouped according to the cell types. Worksheets whose names starting with “Promoters” denotes probes that target promoters, worksheets whose names starting with “SNVs” list probes targeting GWAS variants, worksheets whose names starting with “Intronic Controls” list regions that have no known to date promoter or enhancer annotation and are within intronic regions, worksheets whose names starting with “Intergenic Controls” lists regions that have no known to date promoter or enhancer annotations and are intergenic. Probes labelled as “Intronic Controls” and “Intergenic Controls” are used to produce background interaction frequencies and p-values for the interactions. Three slightly different designs used for the study due to insufficient number of probes in each batch to perform the full experiment. More than 92% of the targeted regions in each probe set overlap, therefore comparable to each other.

Supplementary Table 2. Sequencing statistics and capture efficiencies of HiCap experiments

Supplementary Table 3A, B, C. List of all interactions and their overlap status with chromatin marks and TF binding sites and RNA expression levels of interacting genes in A) AEC and B) ASMC and C) mTHP-1-LPS. In each table, there are four worksheets:

- Worksheet “CellType”_Prom-Prom_Prom-GWAS: Prom-Prom and Prom-GWAS interactions
- Worksheet “CellType”_Prom-Prom_Prom-GWAS WashUGe: Prom-Prom and Prom-GWAS interactions in WashU Epigenome Browser-compatible format (https://epigenomegateway.wustl.edu).
- Worksheet “CellType_Prom-Distal”: Prom-Distal interactions
- Worksheet “AEC_Prom_Distal_WashUGenBrowser”: Prom-Distal interactions in WashU Epigenome Browser compatible format (https://epigenomegateway.wustl.edu).

Prom-Prom and Prom-GWAS interactions can be separated using “Annotation” column header. “Annotation” = 1 stands for promoter-anchored and “Annotation” = 2 stands for GWAS-anchored interaction.

Supplementary Table 4. Gene expression levels in AEC (worksheet AEC_RPKM), ASMC (worksheet ASMC_RPKM) and mTHP-1-LPS (worksheet mTHP-1-LPS_RPKM) cells determined using RNA-Seq method.

Supplementary Table 5. List of ChIP-seq datasets available in ChIP-ATLAS used to overlap distal regions. The content of each worksheet is described below:

HAEC Histone: List of public datasets of H3K27Ac ChIP-Seq datasets performed on HAE cells.

HAEC TF: List of public ChIP-Seq datasets obtained using TF-antibodies in HAE cells

Cardiovascular DNase: public datasets of DNase HS assays performed on cell types classified as "cardiovascular" in ChIP-ATLAS database (chip-atlas.org).

Cardiovascular Histone: public datasets of either H3K27Ac or H3K4me1 ChIP-Seq datasets performed on cell types classified as "cardiovascular" in ChIP-ATLAS database (chip-atlas.org).

Macrophage DNase: public datasets of DNase HS assays performed on primary macrophage cells

Macrophage Histone: public datasets of either H3K27Ac or H3K4me1 ChIP-Seq datasets performed on primary macrophages or monocyte-derived mTHP-1 cells

Macrophage TF: public ChIP-Seq datasets obtained using TF antibodies in either primary macrophages or monocytic or macrophage mTHP-1 cells including activated counterparts.

Supplementary Table 6A, B. A) GWAS variants associated with cardiovascular disease as reported in EBI GWAS Catalogue, B) those targeted in this study. The table includes study, trait, publication and association information for all GWAS variants used in this study.

Supplementary Table 7. Interactions of GWAS variants and those in LD within distal elements found in this study, including GP dataset.

Worksheet AEC_80kb: contains interactions detected in AEC cells

Worksheet ASMC_20kb: contains interactions detected in ASMC cells

Worksheet mTHP-1-LPS_30kb: contains interactions detected in mTHP-1-LPS cells

Variants are also overlapped with relevant chromatin marks and TF binding sites and included in these tables. "Peak Overlap?" column summarises whether the interaction overlap with a relevant peak.

Supplementary Table 8A, B, C, D. Gene ontology enrichment analysis performed using target genes of interacting GWAS variants in A) AEC, B) ASMC, C) mTHP1 cells and D) all combined. Each table also include results using closest gene information.
